# Structurally symmetric near-infrared fluorophore IRDye78-protein complex enables multimodal cancer imaging

**DOI:** 10.7150/thno.54928

**Published:** 2021-01-01

**Authors:** Jiang Yang, Chunhua Zhao, Jacky Lim, Lina Zhao, Ryan Le Tourneau, Qize Zhang, Damien Dobson, Suhasini Joshi, Jiadong Pang, Xiaodong Zhang, Suchetan Pal, Chrysafis Andreou, Hanwen Zhang, Moritz F. Kircher, Hans Schmitthenner

**Affiliations:** 1 State Key Laboratory of Oncology in South China, Collaborative Innovation Center for Cancer Medicine, Sun Yat-sen University Cancer Center, Guangzhou 510006, China.; 2 Department of Radiology, Center for Molecular Imaging and Nanotechnology (CMINT), Memorial Sloan Kettering Cancer Center, New York 10065, NY, USA.; 3 School of Chemistry and Materials Science, Rochester Institute of Technology, Rochester 14623, NY, USA.; 4 CAS Key Laboratory for Biomedical Effects of Nanomaterials and Nanosafety Institute of High Energy Physics, Chinese Academy of Sciences, Beijing 100049, China.; 5 Ph.D. Program in Chemistry, The Graduate Center of the City University of New York, New York 10016, NY, USA.; 6 Department of Chemical Biology, Sloan Kettering Institute, New York 10065, NY, USA.; 7 Department of Physics, Tianjin Key Laboratory of Low Dimensional Materials Physics and Preparing Technology, Tianjin Collaborative Innovation Center of Chemical Science and Engineering, Tianjin University, Tianjin 300354, China.; 8 Department of Chemistry, Indian Institute of Technology Bhilai Raipur, Chhattisgarh 492015, India.; 9 Department of Electrical and Computer Engineering, University of Cyprus, Nicosia 2109, Cyprus.

**Keywords:** renal clearance, carrier proteins, near-infrared fluorophores, structure symmetry, ^89^Zr, breast cancer, glioblastoma, multimodal imaging

## Abstract

**Rationale:** Most contemporary cancer therapeutic paradigms involve initial imaging as a treatment roadmap, followed by the active engagement of surgical operations. Current approved intraoperative contrast agents exemplified by indocyanine green (ICG) have a few drawbacks including the inability of pre-surgical localization. Alternative near-infrared (NIR) dyes including IRDye800cw are being explored in advanced clinical trials but often encounter low chemical yields and complex purifications owing to the asymmetric synthesis. A single contrast agent with ease of synthesis that works in multiple cancer types and simultaneously allows presurgical imaging, intraoperative deep-tissue three-dimensional visualization, and high-speed microscopic visualization of tumor margins *via* spatiotemporally complementary modalities would be beneficial.

**Methods:** Due to the lack of commercial availability and the absence of detailed synthesis and characterization, we proposed a facile and scalable synthesis pathway for the symmetric NIR water-soluble heptamethine sulfoindocyanine IRDye78. The synthesis can be accomplished in four steps from commercially-available building blocks. Its symmetric resonant structure avoided asymmetric synthesis problems while still preserving the benefits of analogous IRDye800cw with commensurable optical properties. Next, we introduced a low-molecular-weight protein alpha-lactalbumin (α-LA) as the carrier that effectively modulates the hepatic clearance of IRDye78 into the preferred renal excretion pathway. We further implemented ^89^Zr radiolabeling onto the protein scaffold for positron emission tomography (PET). The multimodal imaging capability of the fluorophore-protein complex was validated in breast cancer and glioblastoma.

**Results:** The scalable synthesis resulted in high chemical yields, typically 95% yield in the final step of the chloro dye. Chemical structures of intermediates and the final fluorophore were confirmed. Asymmetric IRDye78 exhibited comparable optical features as symmetric IRDye800cw. Its well-balanced quantum yield affords concurrent dual fluorescence and optoacoustic contrast without self-quenching nor concentration-dependent absorption. The NHS ester functionality modulates efficient covalent coupling to reactive side-chain amines to the protein carrier, along with desferrioxamine (DFO) for stable radiolabeling of ^89^Zr. The fluorophore-protein complex advantageously shifted the biodistribution and can be effectively cleared through the urinary pathway. The agent accumulates in tumors and enables triple-modal visualization in mouse xenograft models of both breast and brain cancers.

**Conclusion:** This study described in detail a generalized strategic modulation of clearance routes towards the favorable renal clearance, *via* the introduction of α-LA. IRDye78 as a feasible alternative of IRDye800cw currently in clinical phases was proposed with a facile synthesis and fully characterized for the first time. This fluorophore-protein complex with stable radiolabeling should have great potential for clinical translation where it could enable an elegant workflow from preoperative planning to intraoperative deep tissue and high-resolution image-guided resection.

## Introduction

Thorough pre-surgical localization of cancers followed by precise intraoperative margin visualization allowing complete tumor resections while sparing any healthy tissues and organs would be ideal for the next generation of cancer care. Clinically-approved imaging agents only partially fulfill these goals. ^18^F-fludeoxyglucose is used for pre-surgical PET imaging of cancer with elevated glucose metabolism but is often limited by false positive or false negative results 1. Indocyanine green (ICG) for intraoperative image-guidance with near infra-red fluorescence (NIRF) suffers from low quantum yield (QY), inherent hydrophobicity, and no chemical functionalities to enable bioconjugation. Additionally, its concentration- and environment-dependent optical properties make it suboptimal for multispectral optoacoustic tomography (MSOT), along with an almost exclusive clearance through the slow hepatic-biliary route 2, 3. Ideal oncological imaging agents should satisfy several requirements: (i) enriched and retained in tumors regardless of cancer types, (ii) preferably excreted *via* rapid renal clearance to minimize systemic toxicity, (iii) facilitate both preoperative and intraoperative imaging with sufficient spatial and temporal resolution, and (iv) ideally accomplish these *via* merely one injection of a single multi-modal molecular imaging agent, to increase patient compliance and the chances for regulatory approval.

Longstanding efforts have been made to explore alternative clinically translatable near-infrared (NIR) fluorophores to replace ICG, which is the only FDA-approved fluorochrome thus far. The NIR imaging window allows optimal penetration depth of excitation light and emitted photons due to minimized absorption, scattering, and autofluorescence. The commercially available ICG derivative IRDye800cw was recently used for MSOT to image glioblastoma and pancreatic cancers in mouse models, in addition to NIRF 4, 5, and presented the highest photoacoustic contrast in prostate cancer cells compared to the other four related cyanine dyes 6. However, the inefficient synthesis of asymmetric IRDye800cw is more difficult due to the formation of two symmetrical impurities and undesirable byproducts, therefore requiring extensive purification procedures with subsequent low yields. Fluorophores with symmetric resonance structures are generally less sensitive to the environment with polarity-insensitive emission and sometimes brighter than asymmetric ones 7. Symmetric ZW800 with a heptamethine indocyanine backbone similar to ICG was developed with conjugation functionality, zwitterions, low serum binding, and a QY slightly higher than ICG 8. Another symmetric dye IRDye78 with high water solubility (≥10 mM) in the form of pamidronate and carboxylate was used for osteoblastic and cardiac imaging, respectively 9, 10. However, a facile, cost-effective, and scalable synthesis and characterization towards translation has not been reported and remained a conundrum. It has been previously reported that the symmetric synthesis strategy can be a more efficient and advantageous alternative under certain circumstances over the asymmetric synthesis of NIR fluorophores 11.

Carrier proteins, being naturally zwitterionic, are distinctly advantageous with facilitated transmembrane uptake, high* in vivo* stability, and low inherent toxicity. It has recently been demonstrated that proteins from extracellular matrices are an indispensable amino acid source for cancer cells 12, 13. Fluorophore-protein complexes also have recently been implemented as an integrated platform for cancer imaging and therapy 14-16. However, the complexation has mostly been accomplished through hydrophobic interactions between fluorophores and inner protein cores, which potentially disrupt native protein structures and functions. Such non-covalent binding is also insufficiently stable, and fluorophores could be released during systemic circulation.

Spatially and temporally complimentary imaging modalities with minimally invasiveness through a single agent take advantage of concurrent high resolution, superior sensitivity, and deep tissue penetration and result in improved patient outcome. While the majority of developed agents heavily rely on inorganic nanomaterials such as carbon nanotubes 17, metals 18, metal sulfides 19-21, metal oxides 22, their clinical translation faces crucial challenges due to scale-up capabilities, reproducibility, and toxicity. Organic-based agents have also raised considerable interest for multimodal theranostic applications 23-25, yet toxicity remains a critical issue. Among them, protein-based multimodal theranostic agents are promising to advance into translation against cancers 26.

In this study, we aimed to develop an optimized, inexpensive, facile, and scalable synthesis of the symmetric heptamethine IRDye78 with a batch synthesis of chloro dye in gram scale and a high overall chemical yield, devoid of any asymmetric analog impurities. We then further developed a multi-modal imaging probe for the detection of multiple cancers through covalent in-chain modification of surface lysine residues (Lys) on a small-molecular-weight carrier protein with IRDye78 and the chelator DFO, which confers renal clearance.

## Experimental Section

### Materials

High-purity calcium-depleted bovine α-LA was provided by Agropur. HAuCl_4_·3H_2_O, bovine serum albumin, and cell culture-grade dimethyl sulfoxide (DMSO) were obtained from Sigma-Aldrich. Indocyanine green (ICG) was purchased from Chem-Impex International. Ethylenediamine-N,N,N',N'-tetraacetic acid disodium salt dihydrate (2NA(EDTA·2Na)) and D-luciferin potassium salt were bought from Dojindo Molecular Technologies and Fisher Healthcare respectively. The bifunctional chelator p-isothiocyanatobenzyl-deferoxamine (p-NCS-Bz-DFO) was acquired from Macrocyclics. Ultrapure Milli-Q water with an 18.2 MΩ·cm resistivity was used in the entire study. Sterile pH 7.4 PBS without DNase, RNase, protease, Ca^2+^, and Mg^2+^ was used throughout the study. All other chemicals of analytical grade were acquired from VWR, Sigma-Aldrich, Fisher, and Alfa Aesar and used as received unless otherwise indicated.

### Instrumentation

Nuclear magnetic resonance (NMR) data including ^1^H, ^13^C, and ^1^H-^1^H correlation spectroscopy (COSY) were obtained using a Bruker Avance III 500 MHz NMR spectrometer, and chemical shifts as δ were reported in ppm with tetramethylsilane as the internal standard. Analytical high-performance liquid chromatography (HPLC) with a diode array detector was performed with an Agilent 1100 System. For liquid chromatography-mass spectrometry (LC-MS), a Waters 2695 Alliance HPLC System coupled with a Waters 2998 diode array detector and a Waters 3100 SQ mass spectrometer was employed. An Agilent XDB C18 column, 3.5 μm particle size, in a 3 mm by 100 mm column or a Waters XBridge C18 3.5 μm, 3 mm by 50 mm were used for both LC techniques. The flow rate was 0.5 mL min^-1^ with the solvent gradient starting from 90% solvent A (0.1 M ammonium acetate buffer) and 10% solvent B (acetonitrile or methanol) to 0% solvent A and 100% solvent B at 8 min, or from 20% B to 80% B for the dyes. For diode array analysis, wavelengths from 200-900 nm were collected in the Agilent HPLC and 200-800 nm in the Waters LC-MS. Mass spectral data were collected in both positive and negative modes on a Waters 3100 SQ Mass Spectrometer. High-resolution mass spectrometry data were acquired using a Waters SYNAPT G2-Si high definition mass spectrometry. Conventional flow cytometry was achieved using BD LSR Fortessa^TM^ X-20. Spectral flow cytometry was performed on suspended cells using 638 nm laser with the SONY SP6800 Spectral Cell Analyzer. Zeta potentials of α-LA before and after covalent modification were measured by the Zetasizer Nano ZSE (Malvern Panalytical).

### Synthesis of NIR dye IRDye78: 2,3,3-trimethyl-1-(4-sulfonatobutyl)-3H-indol-1-ium-5-sulfonate (2)

2,3,3-trimethyl-3H-indole-5-sulfonic acid, **(1)** was synthesized from 4-hydrazinobenzenesulfonic acid and 3-methyl-2-butanone as previously described 8. The starting indole **(1)** (2 g, 7.66 mmol) was mixed with n-butyl sultone (4.17 g, 30.7 mmol) and potassium t-butoxide (1.03 g, 9.2 mmol), heated to 120 °C and continuously stirred for 18 h. 50 mL ethyl acetate was added to the reaction to precipitate the product. The solid was collected by vacuum filtration, then triturated in 50 mL refluxing isopropanol and was then cooled, collected, and vacuum dried overnight to obtain 2,3,3-trimethyl-1-(4-sulfonatobutyl)-3H-indol-1-ium-5-sulfonate **(2)** (2.58 g, 6.89 mmol, 89.9% yield). The butyl sulfo indole product was further purified by trituration in boiling ethanol, cooled to ambient temperature, and rinsed with cold ethanol for overnight vacuum drying (2.58 g, 6.89 mmol, 89.9% yield). ^1^H NMR (500 MHz, D_2_O) δ 7.51 (dd, J = 8.1, 2.2 Hz, 1H), 7.42 (d, J = 2.2 Hz, 1H), 6.56 (d, J = 8.3 Hz, 1H), 3.58 (t, J = 6.4 Hz, 2H), 3.50 (t, J = 6.5 Hz, 1H), 3.30 (s, 8H), 3.19-3.09 (m, 1H), 2.97-2.84 (m, 5H), 1.84-1.69 (m, 8H), 1.64 (dt, J = 21.2, 7.1 Hz, 2H), 1.29 (d, J = 8.8 Hz, 4H), 1.07 (s, 3H); LC-MS (ES^-^), calculated for C_15_H_20_NO_6_S_2_: 375.46; LC-MS found [m/z]^-^: 374.08 (M-H)^-^; UV-Vis λ_max_= 270.53.

### 2-((E)-2-((E)-2-chloro-3-(2-((E)-3,3-dimethyl-5-sulfonato-1-(4-sulfonatobutyl)indolin-2-ylidene)ethylidene)cyclohex-1-en-1-yl)vinyl)-3,3-dimethyl-1-(4-sulfonatobutyl)-3H-indol-1-ium-5-sulfonate (4)

The butyl sulfo indole **(2)** (2.58 g, 6.89 mmol) was dissolved in 4 mL water at 30 °C. After the addition of sodium acetate (1.35 g, 13.8 mmol), the mixture was diluted with 15 mL isopropyl alcohol. The chloro-dianil (Sigma-Aldrich), N-((E)-(2-chloro-3-((E)-(phenylimino)methyl)cyclohex-2-en-1-ylidene)methyl)aniline **(3)** (1.11 g, 3.45 mmol), was added, followed by addition of acetic anhydride (1.39 g, 13.8 mmol). The reaction mixture was heated to reflux and stirred for 30 min after which it was cooled to room temperature. The product was collected by vacuum filtration and washed with two rinses of isopropyl alcohol. The damp solid was triturated twice with 20 mL of refluxing methanol, filtered, and dried under vacuum to yield the chloro dye product** (4)** (5.43 g, 6.55 mmol, 95.0% yield). ^1^H NMR (500 MHz, D_2_O) δ 8.12 (d, J = 13.8 Hz, 2H), 7.82-7.73 (m, 2H), 7.69-7.62 (m, 2H), 7.19 (d, J = 8.4 Hz, 2H), 6.06 (d, J = 14.0 Hz, 2H), 4.03 (s, 4H), 2.87 (t, J = 7.4 Hz, 4H), 2.35 (s, 4H), 1.91-1.72 (m, 8H), 1.57 (s, 14H). LC-MS (ES^-^), calculated for C_38_H_48_ClN_2_O_12_S_4_: 886.17008; LC-MS, found [m/z]^-^: 885.56 (M-H), 442.49 (M-2H/2); high-resolution mass spectrometry (HRMS), found [m/z]^-^: 885.1608, [M-H]^-^, 442.0762 (M-2H/2), 294.38207 (M-3H/3); UV-Vis λ_max_= 783.53 from LC-MS DAD in methanol/buffer. Analytically pure samples of chloro dye **(4)** were obtained by preparative chromatography on a C-18 column using a 10-50% acetonitrile-0.02 M trifluoroacetic acid buffer system. The sample was loaded in 0.1 M trifluoroacetic acid. Typically 250 mg of crude dye was chromatographed followed by assaying dye-containing fractions by LC-MS, combining, concentrating, and lyophilizing pure fractions three times, followed by ion exchange to the sodium salt and lyophilization to give the final product, chloro dye **(4).**

### IRDye78 carboxylate (5): (2-((E)-2-((E)-2-(4-(2-carboxyethyl)phenoxy)-3-(2-((E)-3,3-dimethyl-5-sulfonato-1-(4-sulfonatobutyl)indolin-2-ylidene)ethylidene)cyclohex-1-en-1-yl)vinyl)-3,3-dimethyl-1-(4-sulfonatobutyl)-3H-indol-1-ium-5-sulfonate

The chloro dye **(4)** (0.100 g, 0.113 mmol) was dissolved in 2 mL distilled dimethylformamide (DMF) and warmed to 40 °C under Ar atmosphere to dissolve. To a new round-bottom flask was added stripped sodium hydride (0.036 g, 1.51 mmol) and 2 mL DMF chilled in an ice bath under Ar. 4-hydroxy phenyl propionic acid (0.125 g, 0.755 mmol) was added to the second flask followed by warming to room temperature for 10 min. The chloro dye **(4)** in the first flask was then slowly added *via* a syringe. The reaction was stirred for 1 h and intermittently monitored by quenching an aliquot in a mixture of ether and a drop of acetic acid, centrifuging, and dissolving the residue in water followed by analysis by LC-MS. To the reaction mixture was added 20 mL of a solution of 1:1 v/v acetonitrile: ether with 50 μL of glacial acetic acid added to quench the reaction, which was placed overnight at 4 °C to precipitate the product. The crude material was collected by vacuum filtration, washed with ether, and dried under vacuum. The product, **5**, was further purified by reverse phase preparative HPLC on a C-18 column using a 10-50% acetonitrile-0.02 M trifluoroacetic acid buffer system, followed by assaying fractions with LC-MS. The sample was loaded in 0.1 M trifluoroacetic acid. Collected fractions containing the pure product were combined, concentrated, and lyophilized three times, followed by ion exchange to sodium salts. A final lyophilization was carried out to obtain the product as IRDye78 carboxylate **(5)** (0.0815 g, 0.0848 mmol, 75.0% yield). ^1^H NMR (500 MHz, D_2_O) δ 8.11 (d, J = 8.9 Hz, 1H), 7.86 (d, J = 8.6 Hz, 1H), 7.84-7.79 (m, 1H), 7.71-7.64 (m, 4H), 7.59 (s, 3H), 7.14 (t, J = 8.6 Hz, 4H), 6.94-6.80 (m, 2H), 5.92 (d, J = 14.0 Hz, 2H), 3.92-3.77 (m, 4H), 2.81 (t, J = 6.8 Hz, 5H), 2.77-2.68 (m, 2H), 2.54-2.41 (m, 4H), 2.37 (t, J = 7.8 Hz, 2H), 1.80 (s, 2H), 1.76-1.66 (m, 9H), 1.32 (s, 5H), 1.08 (d, J = 3.1 Hz, 13H). LC-MS (ES^-^), calculated for C_47_H_56_N_2_O_15_S_4_: 1017.25635; LC-MS found [m/z]^-^: 1016.04, (M-H), 507.34 (M-2H/2), 337.86 (M-3H/3); HRMS (ES^-^), found [m/z]^-^: 1016.2496 (M-H), 507.1202 (M-2H/2), 337.7436 (M-3H/3); UV-Vis λmax= 775.53 (in LC-MS DAD with methanol/buffer).

### IRDye78-N-hydroxysuccinimide (6): 2-((E)-2-((E)-3-(2-((E)-3,3-dimethyl-5-sulfonato-1-(4-sulfonatobutyl)indolin-2-ylidene)ethylidene)-2-(4-(3-((2,5-dioxopyrrolidin-1-yl)oxy)-3-oxopropyl)phenoxy)cyclohex-1-en-1-yl)vinyl)-3,3-dimethyl-1-(4-sulfonatobutyl)-3H-indol-1-ium-5-sulfonate

IRDye78 carboxylate **(5)** (0.070 g, 0.0728 mmol) was dissolved in 2 mL DMF and heated to 45 °C. N,N,N',N'-tetramethyl-O-(N-succinimidyl)uronium tetrafluoroborate (0.0439 g, 0.146 mmol) was added along with diisopropylamine (0.113 g, 8.74 mmol) and the reaction was allowed to proceed for 30 min with stirring. 10 mL ether was added and the reaction was stored overnight at 4 °C to precipitate. The crude material was collected by filtration, washed with ether, and vacuum dried to derive the NHS ester **(6)** (0.073 g, 0.069 mmol, 95.0% yield). Owing to the high reactivity of NHS esters, the purity was validated through quenching an aliquot by dissolving a trace in 0.01 % butylamine and assaying for the butyl amide derivative using LC-MS. Only IRDye78-NHS ester with >95% purity was used in the study. LC-MS (ES^-^) of butyl amide quenching, calculated for C_51_H_65_N_3_O_14_S_4_: 1071.33, LC-MS found 1071.52 (M-H)^-^, 534.98 (M-2H/2), 356.49 (M-3H/3).

### Computation of electrostatic potential (ESP) charge distributions

The spatial structures of as-synthesized IRDye78-NHS and IRDye800cw-NHS were first optimized using the Gaussian09 software. Calculations were carried out with the generalized gradient approximation (GGA) functional B3LYP. The basis set for Na is LANL2DZ, and the basis set for carbon, hydrogen, sulfur, oxygen, and nitrogen is 6-31G(d). The ESP charge distribution of structures was fitted through the GaussView 5.0 software package.

### Quantum yield measurements

Quantum yield (QY) of IRDye78 was determined in DMSO with ICG (12%) that emits in the NIR window as the fluorescence reference standard 27. UV-vis and steady-state fluorescence spectra were recorded in quartz cuvettes with a SpectraMax M5 multi-detection system (Molecular Devices). Fluorescence for a series of concentrations roughly with optical densities (ODs) of 0.02, 0.04, 0.06, 0.08, and 0.1 was measured. Slopes of integrated fluorescence against absorbance for ICG and IRDye78 were compared to derive the QY of IRDye78 using the following equation:


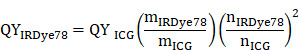


where *m* is the slope and n refers to the solvent refractive index which is DMSO.

### Conjugation of IRDye78 and p-NCS-Bz-DFO to proteins

In this study, we used a one-step reaction for simultaneous covalent in-chain modification of primary amines on Lys side chains of α-LA *via* NHS esters and isothiocyanates to form amide and thiourea respectively. The siderophore-derived chelator DFO was selected for chelating Zr^4+^ because it provides rapid coordination at room temperature at near-neutral pH and stays stable in biologically relevant environments 28. Besides, DFO has been clinically approved to treat metal poisoning and is safe for human use. In brief, IRDye78 and p-NCS-Bz-DFO were dissolved in anhydrous DMSO and added to an α-LA solution in PBS with pH adjusted to 8.5 by K_2_HPO_4_. This pH is sufficient to deprotonate ε-amines of Lys with increased nucleophilicity towards electrophiles. The final DMSO concentration was kept under 10% by volume. Previously, amine landscaping of proteins has shown that fluorescence brightness of conjugated dyes is negatively correlated with reacted amine numbers 29. A threefold molar excess of DFO relative to proteins in optimum is used 30. The reaction mixture containing 500 μM α-LA with a moderate stoichiometry of 1 and 4 molar equivalents for IRDye78 and p-NCS-Bz-DFO was protected from light and allowed to proceed on a rotator at room temperature overnight. The solution was then centrifuged at 6000 rpm for 5 min to remove unreacted p-NCS-Bz-DFO and then washed six times with PBS pH 7.4 in a 3 kDa Amicon^TM^ centrifuge filter unit (EMD Millipore) at 4 °C to remove free IRDye78. The relative surface hydrophobicity was studied using 8-anilinonaphthalene-1-sulfonic acid (ANS). A PBS solution of 100 μg mL^-1^ ANS and 5 μM IRDye78-α-LA-DFO complex was incubated in dark for 1 h at room temperature with fluorescence measured at 360 nm excitation. In the entire study, protein quantification was achieved by the BCA assay (Thermo Scientific) measured at 562 nm. Serum interactions were characterized after 24 h incubation in 50% mouse serum at room temperature by agarose gel electrophoresis (1.5% in 1×TAE running buffer) run at 120 V for 20 min. Steady-state intrinsic tryptophan fluorescence was employed to sensitively probe conformational changes using SpectraMax M5 (Molecular Devices) at the excitation wavelength of 295 nm to eliminate tyrosine fluorescence. Circular dichroism spectroscopy (CD) was recorded on a Chirascan quantitative qCD spectrometer (Applied Photophysics) to study secondary and tertiary structural variations after conjugation in the near- and far-UV windows. PBS was used for baseline subtraction and correction. The mean residue ellipticity θ_m_ was calculated using the formula θ_m_ = θ/(c·n·l), where θ is the recorded ellipticity, c is the molar concentration of proteins, and l is the path length.

### Characterization of oligomerization

To understand the specific biodistribution profile, analytical ultracentrifugation (AUC) was performed using Beckman Coulter Optima^TM^ XL-A analytical ultracentrifuge equipped with the An-50 Ti analytical rotor. The sample solution optimized by OD_280_ together with the buffer control was separately loaded into the double-sector centerpiece with sapphire windows. After temperature equilibrium, the centrifuge was maintained at 50,000 rpm at 20 °C at a radial step size of 0.003 cm for 200 cycle scans, while being monitored simultaneously for absorbance at 280 nm. The buffer density and viscosity at 20 °C were predicted by the Sednterp software and F-statistics were used to determine the root-mean-square deviation (rmsd) within 95% confidence intervals. The sedimentation coefficient c(s) continuous distribution analysis model was employed for sedimentation profiles with numerical Lamm equation solutions using the SEDFIT software. 12% discontinuous native polyacrylamide gel electrophoresis (PAGE) gel was prepared comprised of the stacking gel (0.125 M tri-HCl pH 6.8) and the resolving gel (0.38 M tri-HCl pH 8.8). After electrophoresis, the gel was stained by Coomassie Blue Super Fast Staining Solution (Beyotime Biotechnology) to visualize α-LA oligomerization. For more precise quantification, matrix-assisted laser desorption/ionization time-of-flight mass spectrometry (MALDI-TOF-MS) was carried out using the AB SCIEX TOF/TOF™ 5800 System in a 1:1 v/v mixture of 0.1% aqueous trifluoroacetic acid MALDI matrix and 50 mM sinapinic acid ionization matrix.

### Cell proliferation assay

Cytotoxicity of IRDye78-α-LA-DFO was evaluated using Cell Counting Kit-8 (Dojindo) based on (2-(2-methoxy-4-nitrophenyl)-3-(4-nitrophenyl)-5-(2,4-disulfophenyl)-2H-tetrazolium (WST-8). 1×10^4^ cells/well were seeded in a 96-well plate and pre-incubated for 24 h in a humidified incubator of 5% CO_2_ at 37 °C. The plate was replaced with fresh culture media containing various concentrations of IRDye78-α-LA-DFO and incubated for 24 h in the incubator. 1 mM SDS in media was used as a positive control. 10 μL WST-8 solution was added to each well and incubated for another 4 h in the incubator. The cell metabolic indicator water-soluble formazan dye, which is in proportion to the number of living cells, was measured for absorbance at 450 nm on a microplate reader. Values were normalized to untreated cell controls with blank media subtracted.

### Cell culture

Human breast cancer cell MDA-MB-231 and human glioma cell U-87MG were obtained from American Type Culture Collection (ATCC) and cultured in RPMI and high glucose DMEM media with non-essential amino acids (NEAA) (Core Media Preparation Facility, Memorial Sloan Kettering Cancer Center) which were supplemented with 10% fetal bovine serum (FBS), 2 mM L-glutamine, 1% penicillin and streptomycin in a humidified atmosphere of 5% CO_2_ at 37 °C. U-87MG cells were transduced *in vitro* with a retroviral vector containing Firefly luciferase-IRES-green fluorescent protein (LUC-IRES-GFP) dual reporters and sorted by fluorescence-activated cell sorting (FACS) in the GFP channel based on GFP positivity, as previously described 31. Sorted retrovirally transduced U-87MG cells were further confirmed by wide-field fluorescence microscopy (Nikon). After seeding overnight in cell culture flasks, 10 μM IRDye78 or IRDye78-α-LA-DFO was added with fresh culture media replaced and further incubated for 12 h. Cells were washed three times with PBS and harvested for flow cytometry or cell phantom imaging.

### Animal models of human breast and brain cancers

All animal experiments were performed in accordance with the protocols #06-09-013 and #L102012019002R approved by Institutional Animal Care and Use Committees (IACUC) at Memorial Sloan Kettering Cancer Center and Sun Yat-sen University Cancer Center, respectively, following NIH guidelines for animal welfare. Mice were acquired from the Jackson Laboratory or the Model Animal Research Center (MARC) of Nanjing University. To create breast cancer xenografts, approximately 5x10^6^ MDA-MB-231 human breast cancer cells in a 1:1 medium and Matrigel (Corning) mixture were injected through the thoracic mammary ducts of female athymic J:NU mice of 4-6 weeks. Orthotopic malignant glioma models were created by intracranially injecting 1 μL PBS containing 1x10^5^ luciferase-labeled U-87MG human glioma cells into 4-6 week-old J:NU mice. It was positioned 2 mm anterior, 2 mm right lateral, and 2.5 mm depth with reference to the bregma by a stereotaxic apparatus (David Korf Instruments). The xenograft led to near-complete penetrance of glioblastoma within 2 months. Non-invasive bioluminescence imaging (BLI) was conducted by intraperitoneal injection of 150 mg kg^-1^ D-luciferin to track intracranial glioma progression. For *in vivo* imaging with different modalities, due to the water bath for MSOT as well as radioactivity, a group of different animals (n=3) was used in each case. Gas anesthesia of vaporized 2% isoflurane in O_2_ flows was maintained in all imaging procedures and CO_2_ inhalation was applied for euthanasia. Female C57BL/6J mice of 10-12 weeks were used for biodistribution. Organs of interest were collected at different time points following tail-vein injection and subject to *ex vivo* imaging. Regions of interest for organs were specified by the free-drawn function and guided by white-light photographs for quantification. Renal clearance was studied also in C57BL/6J mice by collecting excreted urine 3 h after injection.

### MRI imaging

Glioblastoma incidence 4 weeks post-implantation was also monitored by subsequent magnetic resonance imaging (MRI) using a 7 Tesla small animal MRI scanner (Bruker Biospin) equipped with a 640 mT m^-1^ and 4600 T m^-1^ s^-1^ slew rate 12 cm ID gradient coil. A custom-built 32 mm quadrature RF body coil (Starks Contrast MRI Coils Research) was used for RF excitation and detection with Bruker Avance electronics. Coronal 2D T_2_-weighted fast spin-echo rapid acquisition with relaxation enhancement (RARE) sequence was applied at 1 mm slice thickness, 180° flip angle, 1.5 s TR, 50 ms TE and 256 × 160 matrix.

### Near-infrared fluorescence imaging

IVIS Spectrum imaging system (PerkinElmer) was used for non-invasive *in vivo* near-infrared fluorescence imaging (NIRF). 20 and 50 nmol of IRDye78-α-LA-DFO in 200 μL PBS were injected into the lateral tail veins of J:NU xenograft mice inoculated with MDA-MB-231 breast tumors and U-87MG brain tumors respectively. Living Image software (PerkinElmer) was used for image analysis and fluorescence multispectral unmixing was performed to differentiate IRDye78 specific signals from autofluorescence when applicable. Mean signal intensities for regions of interest (ROI) were computed for tumor areas identified by bright fields. All fluorescence images were thresholded for optimal tumor display. For intraoperative NIRF image-guided surgery, mice bearing MDA-MB-231 tumors were surgically exposed 24 h post-injection, and thirds of it were stepwise resected until complete removal was achieved as confirmed by visual scrutiny and absence of remaining signals on the resection bed (n=2). Intraoperative imaging was acquired using a Bruker FX PRO system.

### MSOT imaging

Optoacoustic measurements were conducted in a 34 °C water chamber on an inVision MSOT system (iThera Medical), under the excitation of a Q-switched Nd:YAG laser (pulse duration ≤10 ns and repetition rate=10 Hz) ranging from 680 to 900 nm. Homogeneous illumination was delivered from ten output arms of a fiber bundle at a 13° angle to the imaging plane. A cylindrically focused 256-element ultrasound transducer array covering 270 degrees with a 5 MHz center frequency allows the acquisition of traverse-plane images. 2D images were reconstructed in the ViewMSOT software (iThera Medical) using the standard back-projection algorithm. Linear spectral unmixing was then performed on a pixel-by-pixel basis with negative values discarded and fit the measured optoacoustic spectra according to corresponding predefined absorption spectra such as hemoglobin (Hb), oxyhemoglobin (HbO_2_), and IRDye78-α-LA-DFO. 3D tomographic images were reconstructed by interpolated model-matrix inversion. *In vitro* imaging was carried out in a 20 mL pre-molded syringe phantom comprised of 1.5% w/w agarose, 0.002% v/v India black ink, and 1.2% v/v 20% intralipids to mimic tissue scattering and absorption. Photostability was investigated *via* continuous irradiation by a pulse laser constantly sweeping from 680 to 900 nm for 2 h. For *in vivo* imaging, the injection dose was the same as for NIRF, and a single-wavelength optoacoustic image at 680 nm was used as the background of anatomical reference.

### Radiolabeling IRDye78-α-LA-DFO with ^89^Zr

^89^Zr (t_1/2_=78.4 h) was produced in the form of ^89^Zr-oxalate from an on-site TR19/9 cyclotron (Ebco Industries) at Memorial Sloan Kettering Cancer Center *via* the ^89^Y (p,n)^89^Zr nuclear reaction using a 100% naturally abundant ^89^Y target and purified with a specific activity of 5.28-13.43 mCi μg^-1^. A CRC-15R Dose Calibrator (Capintec) was used to measure activities. The ^89^Zr-oxalate solution was neutralized to pH 7 by adding 1.0 M Na_2_CO_3_, monitored using pH test strips. Radiolabeling reaction was carried out by adding the pH-adjusted ^89^Zr-oxalate to 500 μM IRDye78-α-LA-DFO in pH 7.4 PBS and kept agitated at room temperature for 60 min on a heating block at 600 rpm. The reaction mixture was then quenched by adding 5 μL 50 mM pH 5.5 EDTA. The radiolabeling procedure is robust with typical yields >90% for the crude mixture. Radiochemical purity was verified by spotting a 1 μL sample (<1 μCi) on silica gel-impregnated instant thin layer chromatography (iTLC) strips (Agilent Technologies) with 50 mM pH 5.5 EDTA as eluent and then scanned using Bioscan AR-2000 radio-TLC imaging scanner. The radio-labeled complex was further purified by washing with pH 7.4 PBS using a 3 kDa molecular weight cut-off centrifuge filter until no ^89^Zr radioactivity was detected in the filtrate. Serum stability of IRDye78-α-LA-DFO-^89^Zr (~50 μCi) was investigated over time at 37 °C in 50% mouse serum (Sigma-Aldrich) on a thermomixer at 300 rpm, followed by iTLC analysis. The challenge test was conducted in the presence of 1 mM EDTA for 2 h at room temperature. Freshly prepared IRDye78-α-LA-DFO-^89^Zr was immediately used for *in vitro* and *in vivo* studies to minimize potential radiolysis.

### PET imaging

J:NU mice with MDA-MB-231 breast tumors or U-87MG brain tumors were intravenously (*i.v.*) injected with 200 μL 400-500 μCi (14.8-18.5 MBq) radiolabeled IRDye78-α-LA-DFO-^89^Zr or free ^89^Zr and imaged on a Focus 120 small-animal microPET scanner (Concorde Microsystems) at predetermined time points. The static PET scans typically recorded a minimum of 40 million coincident events in a duration of 15 min. An energy discrimination window of 350-700 keV and a coincidence timing window of 6 ns was applied. Image data were sorted by Fourier rebinning into 2D histograms and reconstructed by back-projection for the transverse images. Normalization was performed to correct non-uniform PET response, dead-time count losses, positron branching ratio, and physical decay to the time of injection, without applying attenuation, scatter, or partial-volume averaging. Visualization of images was carried out using ASIPro VM software (Concorde Microsystems).

### Histology

Freshly dissected brain tissues were subject to a fixed frozen preparation procedure. Briefly, tissues were fixed in 4% paraformaldehyde in PBS overnight, left in 30% w/w sucrose in PBS overnight, and incubated in Tissue-Tek^®^ O.C.T. compounds (VWR) for 4 h on ice. Tissues were then embedded in fresh O.C.T. in cryomolds under 2-methylbutane bath on dry ice. Ten-micrometer sections were sliced on a Leica CM1950 Clinical Cryostat (Leica Biosystems) at -20 °C. *Ex vivo* NIR fluorescence imaging was performed on the Odyssey imager (Li-Cor). Hematoxylin and eosin (H&E) stained sections were scanned on a Mirax digital slide scanner (Zeiss) and visualized in Pannoramic Viewer software (3DHistech).

### Digital autoradiography

Fixed frozen tissue sections were underexposure at -20 °C to phosphor-imaging plates (Fujifilm BASMS2325, Fuji Photo Film, Japan). The imaging plates were then read using a Typhoon FLA 7000 IP imager (GE Healthcare Life Sciences) in 25 μm resolution to generate digital autoradiograms that were processed in ImageJ (NIH).

### Immunofluorescence staining

Immunofluorescence (IF) staining was performed on the Discovery XT processor (Ventana Medical Systems) on 10 μm frozen sections of GFP-LUC transduced U-87MG gliomas. After decay for 10 half-lives of ^89^Zr, antigen retrieval from cryosections was accomplished with CC1 buffer (Ventana Medical Systems) and blocked for 30 min with Background Buster solution (Innovex). Slides were incubated in avidin-biotin blocking (Ventana Medical Systems) for 8 min, followed by 2 μg mL^-1^ chicken polyclonal anti-GFP (ab13970, Abcam) for 5 h. Biotinylated goat anti-chicken IgG secondary antibody (Vector Labs) at 1:200 dilution and a combination of streptavidin-HRP D (DAB Map Kit, Ventana Medical Systems) and Tyramide Alexa Fluor 488 (Invitrogen) at manufacturer's recommended predetermined dilutions were used to detect anti-GFP primary antibodies. 10 min incubation of 5 μg 4',6-diamidino-2-phenylindole (DAPI) was conducted as nuclear counterstains, followed by the addition of Mowiol for mounting coverslips for scanning.

### Statistical analysis

Data were presented as mean ± standard deviation. Differences of mean signals were statistically tested using the two-tailed paired Student's t-test unless indicated otherwise. A minimum of three animals per group was used in the study.

## Results and Discussion

**Figure 1:**
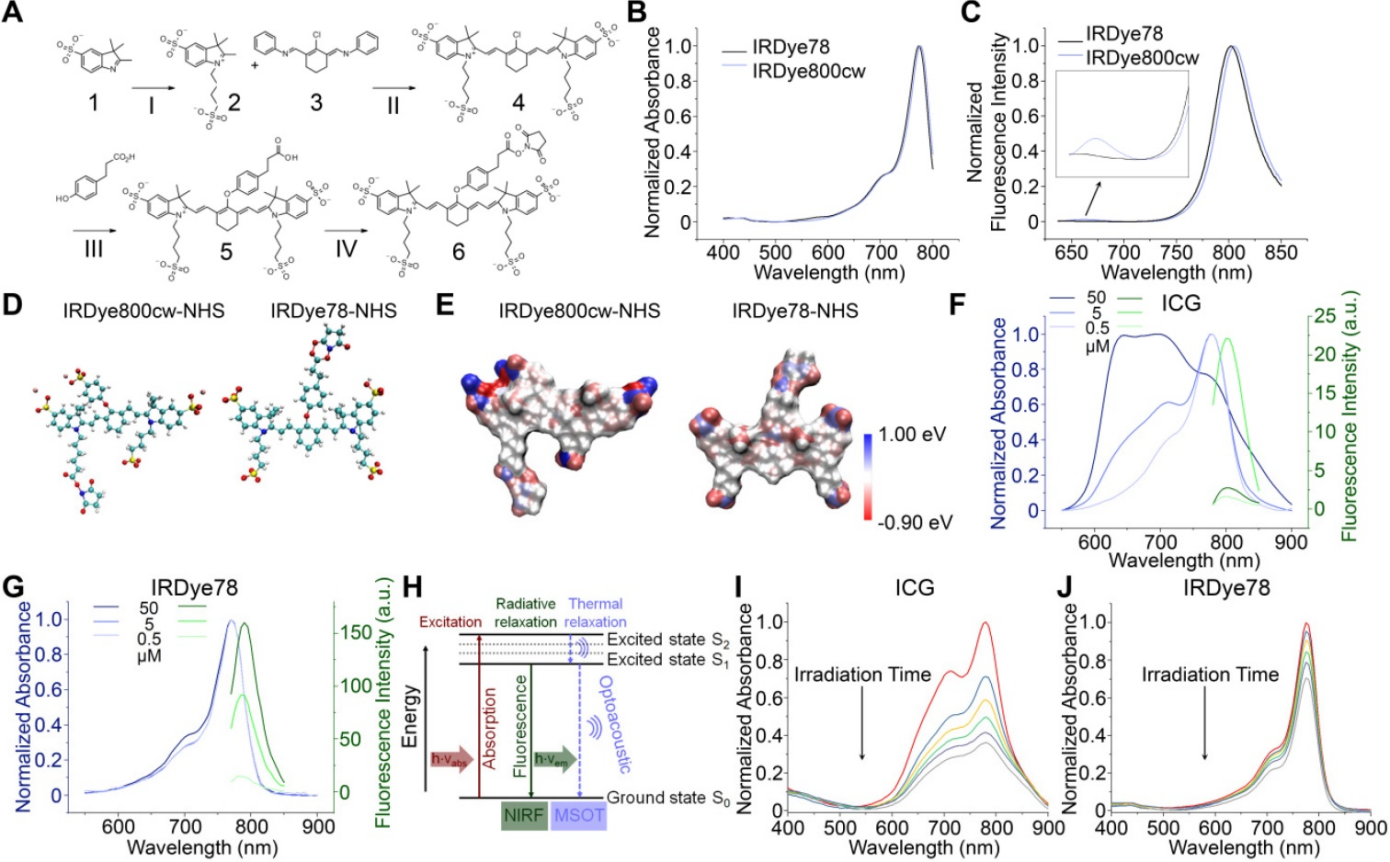
Synthesis, characterization and comparisons of IRDye78. (A) Organic synthesis route of structurally symmetric amine-reactive NIR dye IRDye78. I: K^+^OtBu^-^, n-butyl sultone; II: Na^+^OAc^-^, IPA; III: NaH, DMF; IV: TSTU, DMF. (B) UV-vis absorption and (C) fluorescence spectra of structurally symmetric IRDye78 and asymmetric IRDye800cw. (D) Optimized geometric molecular structures. (E) ESP charge distribution. UV-vis absorption and fluorescence spectra of (F) ICG and (G) IRDye78 in PBS. (H) Principle of for simultaneous NIRF and MSOT imaging. Upon excitation, NIR dyes undergo both radiative and non-radiative energetic transitions that generate fluorescence and acoustic signals, respectively. UV-vis spectra of 5 µM (I) ICG and (J) IRDye78 after 0, 1, 3, 5, 7, and 10 min of irradiation with an 808 nm NIR laser (0.1 W/cm^2^).

We first derived the bis-sulfonated indole **2** from mono-sulfonated indole **1** (**Figure 1A**) 8, 32. Four-carbon sulfonate side chains were employed for increased stability and solubility in both organic and aqueous solvents to facilitate synthesis and bioavailability. The symmetric chloro intermediate **4** was directly formed through a one-step condensation reaction of **2** and chloro-dianil building block **3**. IRDye78 carboxylate **5** was obtained *via* the reaction of sodium hydride with a displacement of the chloro group by phenyl propionic acid and purified by preparative reversed-phase high-performance liquid chromatography (HPLC). The NHS ester of IRDye78 **6** facilitating subsequent biomolecular conjugation was produced by reacting carboxylate **5** with *N*,*N*,*N'*,*N'*-tetramethyl-*O*-(*N*-succinimidyl)uronium tetrafluoroborate (TSTU) in the presence of *N*,*N*-diisopropylethylamine (DIPEA), yielding 99% chemical purity after precipitation, as assayed by a butyl-amine quenched derivative. All chemical structures and corresponding purification of all intermediates, as well as the final products, were confirmed by ^1^H NMR, 2D double-quantum filtered ^1^H-^1^H correlation spectroscopy (DQF-COSY), LC-MS, and HRMS (**Figure S1-S9** and **Table S1-S2**). This symmetric synthesis essentially circumvents asymmetric problems involving both symmetric impurities, half dyes, and hydrolyzed half dyes impurities, resulting in high product yields. The ease of synthesis, reduced cost, high chemical yields, and scale-up capability render it promising for potential clinical translation as a handy alternative fluorophore. The entire synthesis of IRDye78, as summarized in **Figure 1A**, can be achieved in just four steps and it only requires simple purification, devoid of asymmetric impurities. We placed the NHS ester group in the center to allow biomolecular conjugation. The four sulfonic acid groups evenly distributed over the molecule essentially shield the hydrophobic core and increase its solubility. Symmetric IRDye78 presents minor hypsochromic shifts in absorption and emission peaks, compared to the asymmetric counterpart IRDye800cw (**Figure 1B-C**). The positive charge in IRDye78 **6** is delocalized in the entire symmetric molecule by tautomerism without preference on individual indole N atoms (**Figure 1D-E**) and does not present dichromic fluorescence (**Figure 1C**). Structurally asymmetric IRDye800cw NHS ester bears undesirable secondary fluorescence (**Figure 1C**), due to partially localized non-equivalent charge distribution as calculated by electrostatic potential (ESP) charge distribution (**Figure 1E** and **S10**), similar to that observed from ICG and ICG-NH_2_ 33. The secondary emission is diminished in IRDye78 due to evenly delocalized positive charge from nitrogen by tautomerism.

The characteristic absorption of clinically-used ICG is widely known to be heavily influenced by the local dye concentration because of fluorescence self-quenching from aggregation at high concentrations (**Figure 1F**), resulting from its poor solubility under physiological conditions and the high degree of absorption-emission spectral overlap 34. This phenomenon raises major challenges for both NIRF and MSOT imaging. Conversely, IRDye78 has neither apparent absorption variation nor self-quenching (**Figure 1G**), with a QY (24%) two-fold of ICG (12%) (**Figure S11**) 27. Such moderate QY and unambiguous absorption and emission spectra are optimal for simultaneous NIRF and MSOT imaging. In this well-matched fluorophore, excited electrons in IRDye78 can relax in both radiative and non-radiative manners for NIRF and MSOT imaging, respectively (**Figure 1H**). Moreover, IRDye78 is also less vulnerable to photobleaching with higher stability than ICG (**Figure 1I-J** and **S12**).

**Figure 2:**
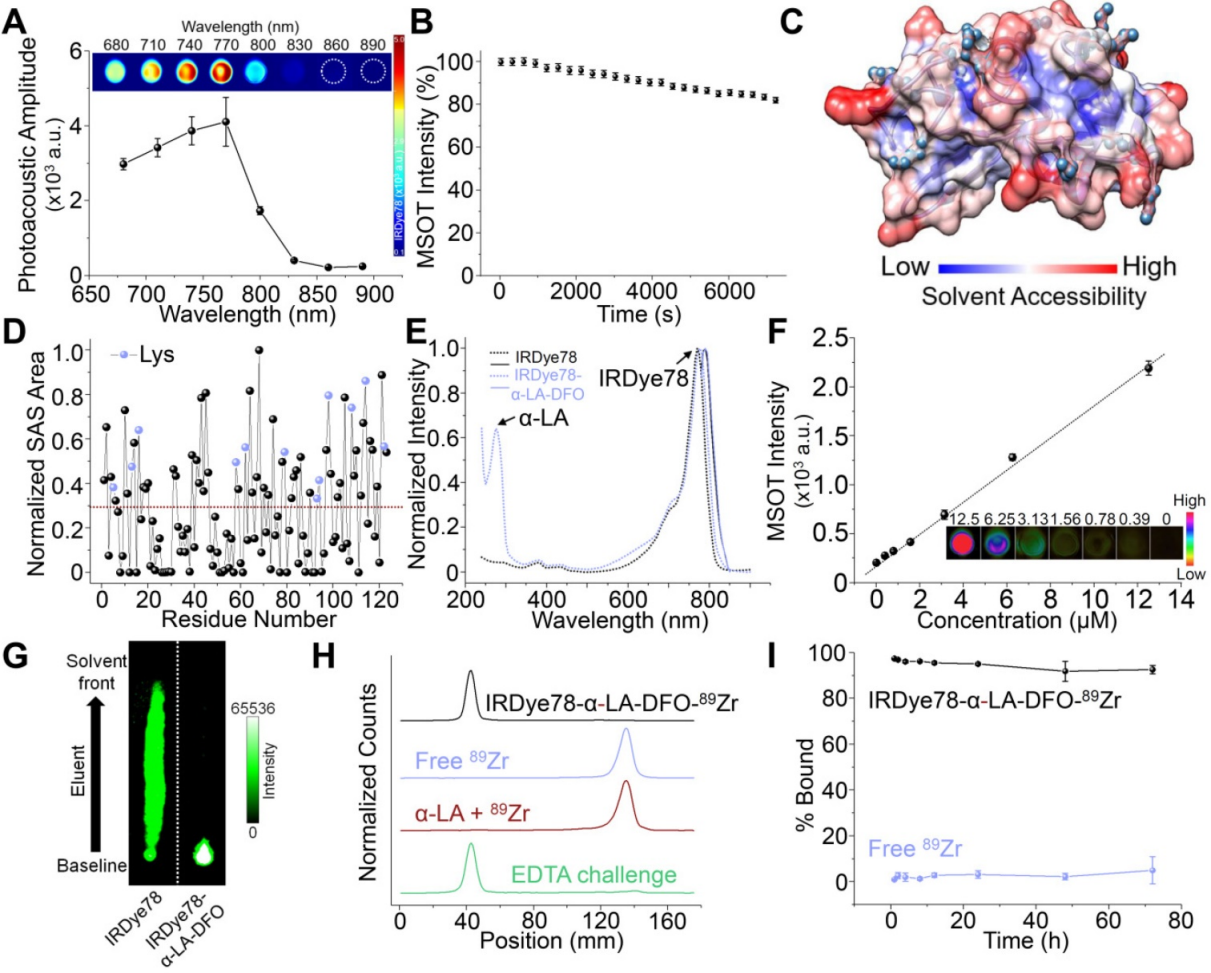
Conjugation and characterization of IRDye78-α-LA-DFO. (A) Photoacoustic spectrum of IRDye78. Inset shows single-wavelength photoacoustic images. (B) Multispectral optoacoustic stability of IRDye78. (C) Surface map of solvent accessibility for α-LA. Lys residues were denoted as cyan balls. (D) The solvent-accessible surface area of all amino acid residues in α-LA normalized using a generalized empirical approach previously proposed^42^. (E) Absorption (dotted lines) and fluorescence (solid lines) spectra of IRDye78 and IRDye78-α-LA-DFO. (F) MSOT intensities of IRDye78-α-LA-DFO phantoms. Inset shows unmixed MSOT images. (G) NIRF images of iTLC. (H) iTLC at room temperature. ^89^Zr^4+^ bound to the IRDye78-α-LA-DFO appeared at the baseline origin. (I) Serum stability of free ^89^Zr and radiolabeled IRDye78-α-LA-DFO-^89^Zr at 37 °C.

The *in vitro* photoacoustic spectrum of IRDye78 closely resembles its absorption spectrum with the maximum photoacoustic amplitude at 770 nm (**Figure 2A**). IRDye78 displayed an acceptable MSOT photostability upon continuous multispectral irradiation (**Figure 2B** and **Video S1**) and maintained stable fluorescence intensities over the physiological pH range and at various temperatures (**Figure S13A-B**). It shows higher fluorescence intensities in a variety of organic solvents than in aqueous solution (**Figure S13C**).

Through analysis of Lys solvent accessibility, mammalian milk-derived α-LA was identified as a novel building block for multi-modal imaging, given its large Lys solvent accessible surface (SAS) area and a high percentage of reactive Lys that thermodynamically allows chemical conjugation (**Figure S14**). Most Lys residues in α-LA are positioned on a highly accessible surface with a large specific SAS area among all residues (**Figure 2C-D**) and rendered a high labeling efficiency, as observed by the minimal amount of free dyes in the filtrate (**Figure S15**). Moreover, α-LA has been officially labeled “generally regarded as safe” (GRAS) by the US FDA (Notice 763). The optical properties of IRDye78 remained largely unaltered in the complex with the characteristic peak of α-LA centered around 280 nm (**Figure 2E**). The complex displayed good concentration-dependent MSOT linearity (R^2^=0.995) (**Figure 2F**), more stable fluorescence in serum, and weaker serum interactions than its small molecule counterpart (**Figure S16**), as well as minimal cytotoxicity (**Figure S17**). Surface hydrophobicity of α-LA decreased after covalent conjugation (**Figure S18A**), which was attributed to the hydrophilic sulfonates and DFO, while the zeta potential was still retained at a well-balanced level of <-10 mV (**Figure S18B**). The protein conformation in the light of secondary and tertiary structures was also preserved as observed by intrinsic tryptophan fluorescence spectra, near- and far-UV CD spectra (**Figure S18C-E**). The preservation of the native protein conformation without significant structural variations was attributed to the low stoichiometric labeling ratio and mild labeling conditions. Small molecular IRDye78 eluted freely towards the solvent front with iTLC, but IRDye78-α-LA-DFO complex was retained at the baseline (**Figure 2G**). Free ^89^Zr isotope was barely bound to α-LA, whereas the ^89^Zr-chelated complex showed high radiochemical purity and did not dissociate upon EDTA challenge (**Figure 2H** and **S19**). Such ^89^Zr coordination by IRDye78-α-LA-DFO was also highly stable in serum over 72 h at 37 °C, while free ^89^Zr exhibited low serum binding (**Figure 2I** and **S20**).

**Figure 3:**
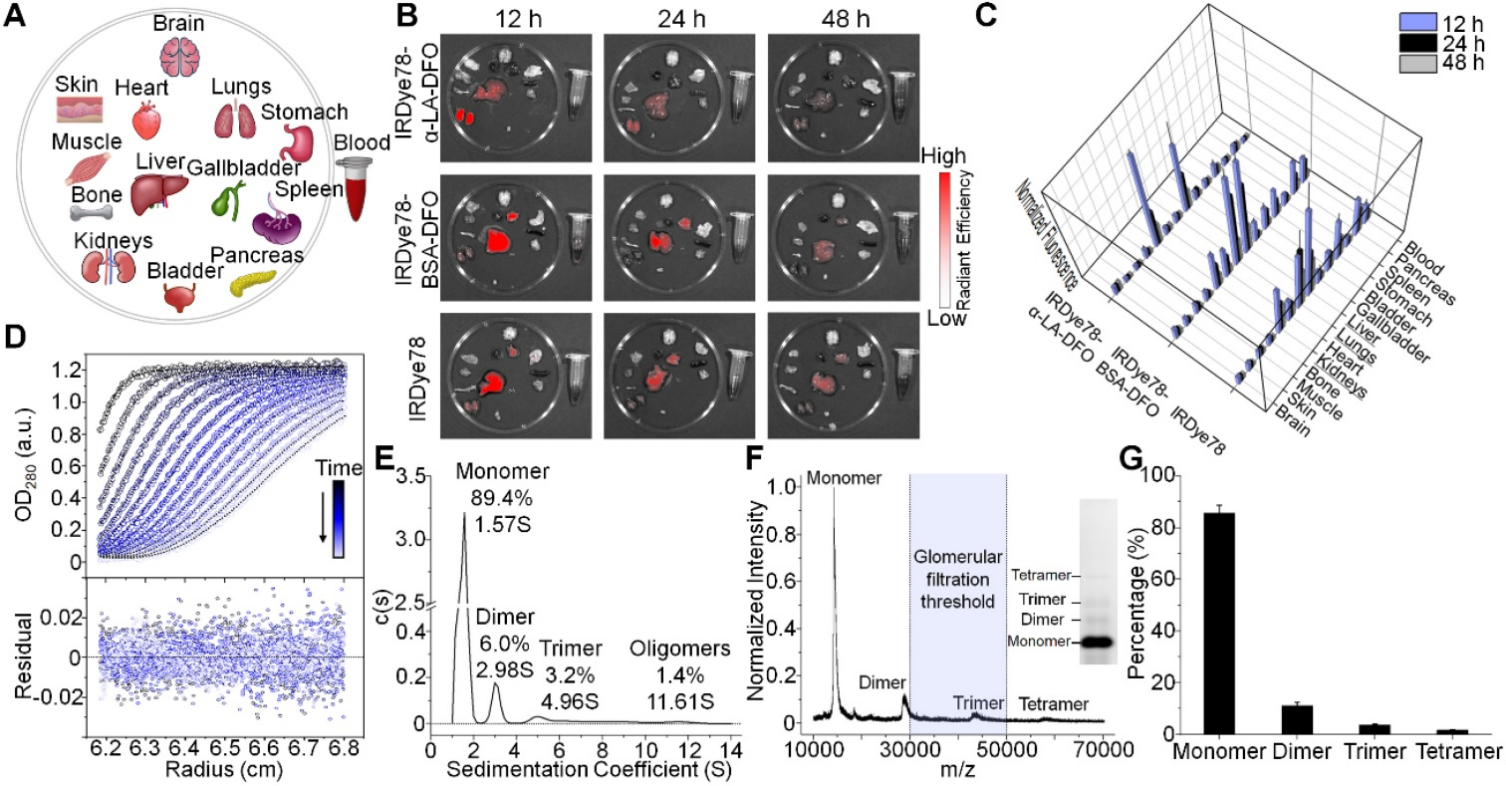
IRDye78-α-LA-DFO shifted the excretion route towards renal clearance. (A) Schematic organ layout for biodistribution. (B) *Ex vivo* fluorescence imaging following euthanasia at 12, 24, and 48 h after systemic administration. (C) Corresponding distribution in organs of interest (n=3). (D) Analytical ultracentrifugation (AUC) of α-LA. Sedimentation velocity profiles with best-fit numerical Lamm equation solutions are shown as dotted lines (upper panel). An rmsd of 0.0078 OD is obtained for residuals (lower panel). (E) Sedimentation coefficient distribution showing proportions of monomers (1.57 S), dimers (2.98 S), trimers (4.96 S) as well as high-molecular-weight oligomers and aggregates (11.61 S). (F) MALDI-TOF-MS showing co-existence of monomers, dimers, trimers, and tetramers. The highlighted region represents the renal clearance molecular weight threshold. Native PAGE gel (inset) confirms the four populations of oligomerization. (G) Relative abundance of monomeric and multimeric species quantified from MALDI-TOF-MS.

**Figure 4:**
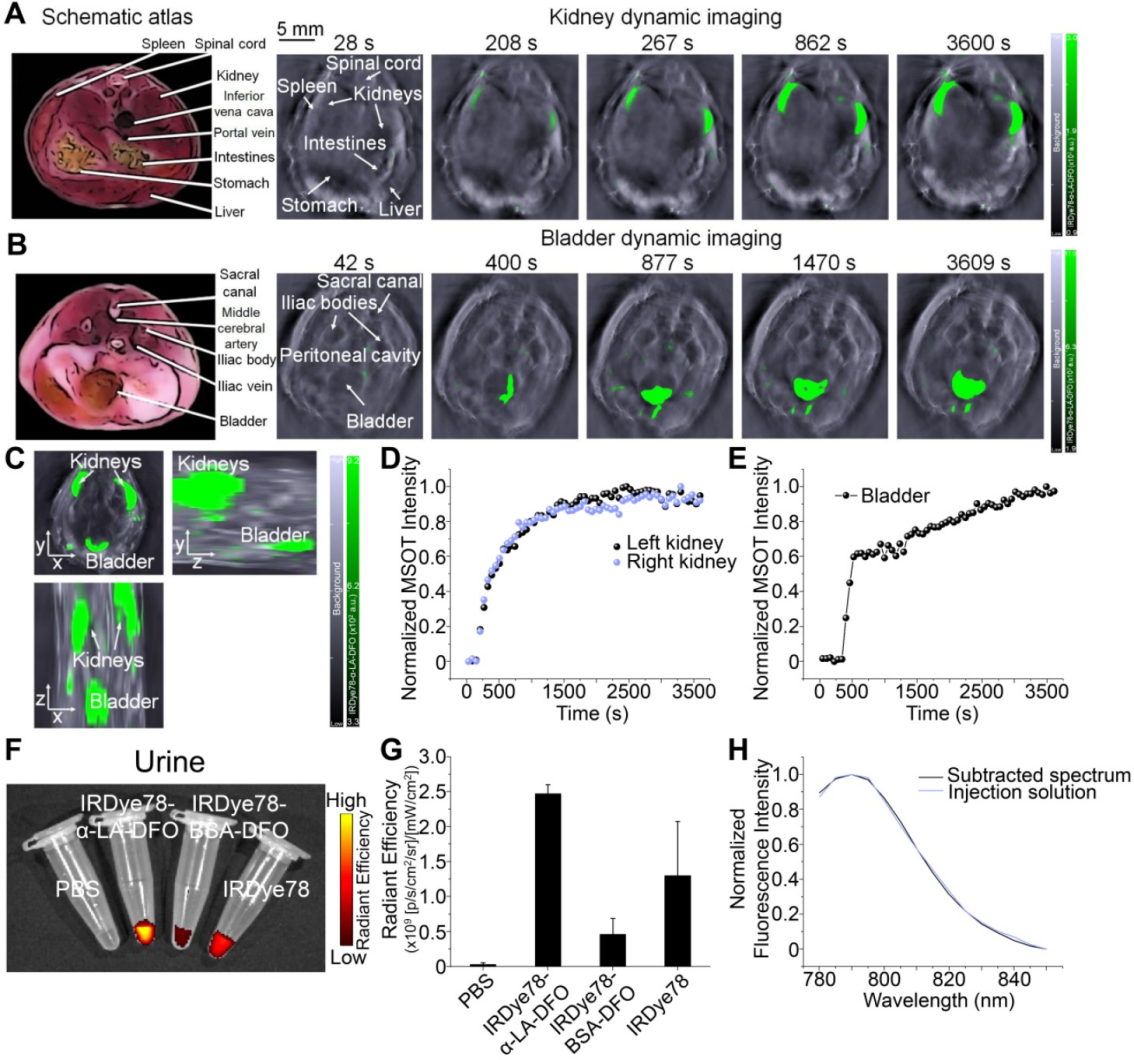
Imaging of renal clearance. (A) Real-time MSOT dynamic imaging for evaluation of kidneys of a healthy J:NU mouse following injection of IRDye78-α-LA-DFO. (B) Time-course accumulation profile of IRDye78-α-LA-DFO in the bladder imaged by MSOT. Schematic atlas reference slices were shown for anatomical guidance. (C) A representative static 3D tomographic MSOT scan of the mouse urinary system in three dimensions imaged 1 h after systemic injection. Dynamic MSOT intensities in (D) both kidneys and (E) the bladder after intravenous injection. (F) Representative NIRF images of urine collected from C57BL/6J mice 3 h after injection. (G) Corresponding signal quantificaiton of collected urine. (H) Spectral features of IRDye78-α-LA-DFO remain identical in the pre-injection solution and the excreted urine (derived from Figure S22).

IRDye78 in the NHS ester form was primarily distributed in the liver and lungs with minor signals in kidneys, following similar patterns (but lower uptake in the lungs) as the IRDye78-BSA-DFO complex (**Figure 3A-C**). By tailoring the molecular assembly with α-LA, the clearance of IRDye78-α-LA-DFO shifted from the hepatic to the more rapid renal pathway, with minor liver uptake due to the coexistence of high-molecular-weight multimers beyond the glomerular filtration threshold as detected by AUC (**Figure 3D-E**), native PAGE, and MALDI-TOF-MS (**Figure 3F-G**) 35. Monomers and the majority of dimers are within the molecular-weight renal clearance threshold (30-50 kDa), while higher degrees of multimers are subject to slower hepatic clearance. Next, we used MSOT imaging to probe deeper anatomical features to track excretion in real-time (**Video S2-S3**). IRDye78 specific signals were observed to be enriched in the urinary system of kidneys and bladder within a short time of <200 s post-injection (**Figure 4A-C**). The bladder and both kidneys as well as nearby lymphatic systems can be clearly identified. *In vivo* accumulation kinetics similar for both kidneys reaching a plateau around 1750 s, while MSOT signals in the bladder increased over time due to continuous clearance (**Figure 4D-E**). The left and right functional healthy kidneys also demonstrated similar *ex vivo* MSOT signals (**Figure S21**). Meanwhile, a significantly elevated excretion into urine was expectedly observed (**Figure 4F-G**) and the fluorescence spectrum of IRDye78-α-LA-DFO remained unchanged after excretion into urine, showing its high stability (**Figure 4H** and** S22**).

**Figure 5:**
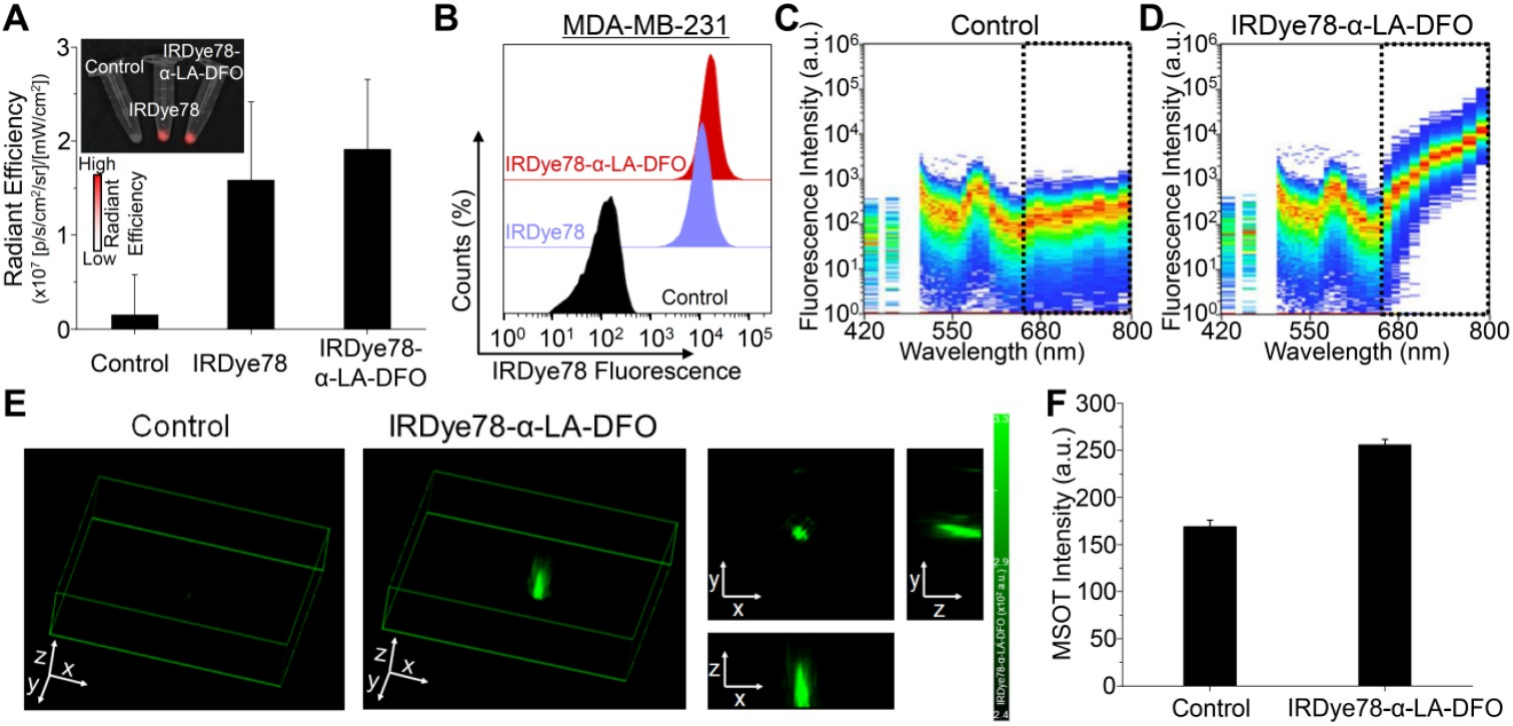
Cellular uptake. (A) NIRF images of pelleted phantoms of MDA-MB-231 cancer cells following 12 h uptake of IRDye78 or IRDye78-α-LA-DFO. (B) Flow cytometry of MDA-MB-231 cells after 12 h uptake of IRDye78-α-LA-DFO (red) or IRDye78 (blue). (C, D) Spectral flow cytometry showing an obvious increase in the NIR region (dotted lines) in MDA-MB-231 cells with IRDye78-α-LA-DFO uptake. (E) MSOT imaging of MDA-MB-231 cell phantoms. The 3D reconstructed image and 2D MSOT images in all three dimensions outlined the pelleted shape of the cell phantom with specific MSOT signals. (F) Cells after uptake of IRDye78-α-LA-DFO exhibited markedly higher MSOT intensity.

**Figure 6:**
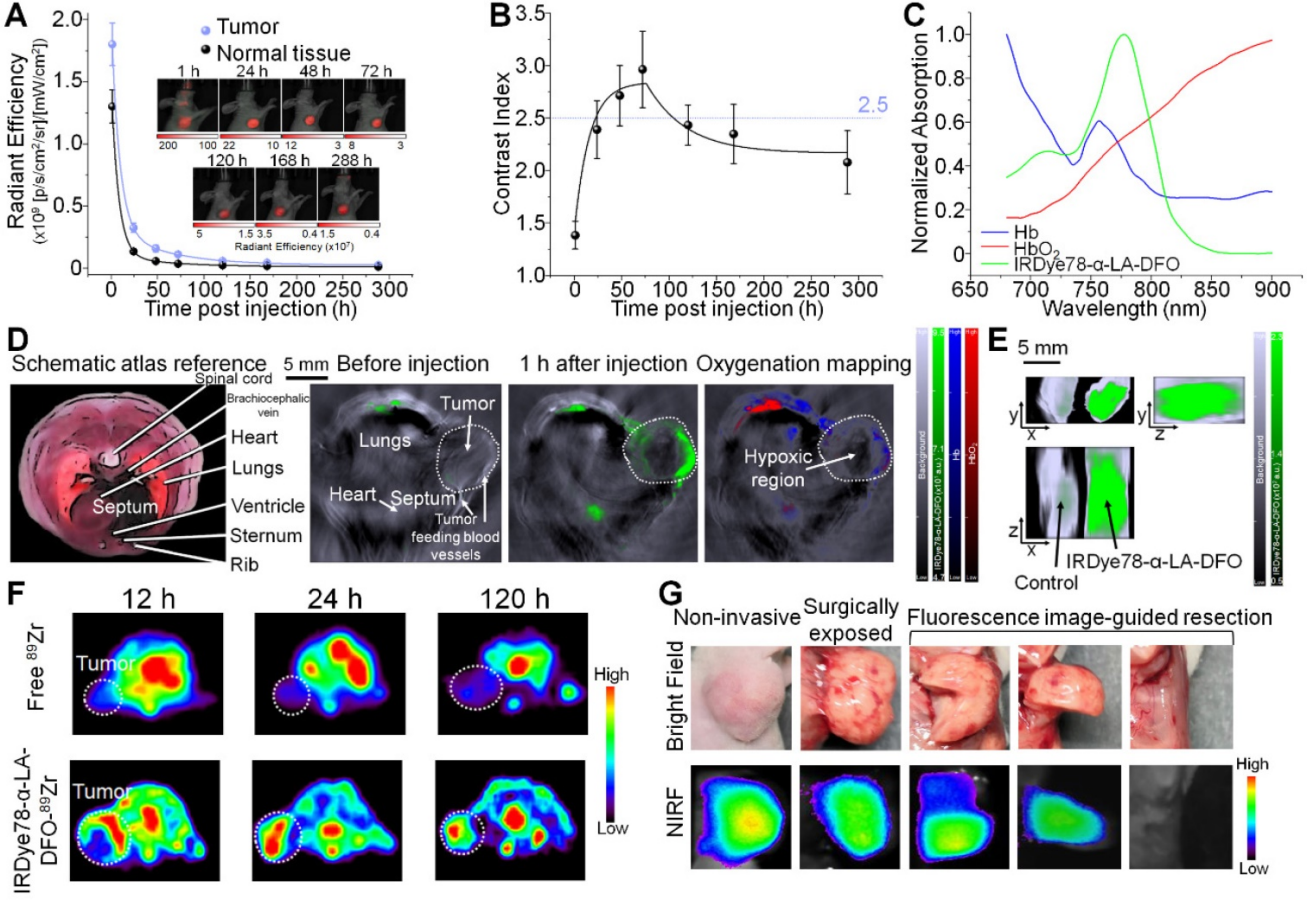
Non-invasive *in vivo* imaging of MDA-MB-231 breast tumors by multiple modalities. (A) Tumor retention kinetics of IRDye78-α-LA-DFO. Inset: representative NIRF images at reported time points post* i.v.* injection. IRDye78-α-LA-DFO and autofluorescence were coded in red and green and superimposed on bright-field images. (B) Contrast index of IRDye78-α-LA-DFO as a function of *p.i.* time. (C) Absorption spectra of major endogenous interfering Hb and HbO_2_ in comparison to the exogenous IRDye78-α-LA-DFO in the NIR imaging window. (D) MSOT images of breast tumors before and after *i.v.* administration of IRDye78-α-LA-DFO. A multispectrally resolved oxygenation image denoting Hb and HbO_2_ distribution showed interior tumor necrosis with hypoxia. An atlas scheme was shown as anatomical reference. (E) A representative *ex vivo* MSOT image of breast tumors resected from xenograft mice in three dimensions. (F) Representative axial PET images of breast tumors at different time points *p.i.* of free ^89^Zr or IRDye78-α-LA-DFO-^89^Zr. Tumor regions were highlighted as white dotted circles. (G) Stepwise intraoperative NIRF-image guided surgery for a breast tumor 24 h* p.i.*

IRDye78-α-LA-DFO illustrated efficient *in vitro* uptake in cancer cells as revealed by the strong fluorescence signal as in imaging phantoms and flow cytometry (**Figure 5A-F**). Surprisingly, IRDye78-α-LA-DFO has an insignificantly higher signal than IRDye78 (**Figure 5A-B**), possibly owing to the elevated uptake of extracellular proteins by cancer cells as a major metabolic pathway 13. MDA-MB-231 human breast tumors in xenograft mouse models were distinctly delineated from surrounding tissues 1 h following *i.v.* administration due to different vascularity and the enhanced permeability and retention (EPR) effect 36. Superior and sustained accumulation of IRDye78-α-LA-DFO was observed in tumors that remained distinguishable for 288 h with a tumor retention half-life of 40.05±4.77 h compared to that of normal tissues of 34.34±6.48 h (**Figure 6A**). The fluorescence imaging contrast index of IRDye78-α-LA-DFO exceeded the diagnostic threshold of 2.5 after 48 h and reached a maximum of 3.0 at 72 h post-injection (*p.i.*) 37 (**Figure 6B**). Through* in vivo* multispectral unmixing, IRDye78-α-LA-DFO can be specifically differentiated from major intrinsic biological absorbers of Hb and HbO_2_ (**Figure 6C**), revealing tumor accumulation, blood circulation in the heart, and hypoxic tumor microenvironment with a high degree of clarity by MSOT imaging (**Figure 6D-E** and **S23-24**). The radioactive complex IRDye78-α-LA-DFO-^89^Zr also showed higher and prolonged tumor retention compared to free ^89^Zr in MDA-MB-231 breast tumors, as revealed *via in vivo* PET imaging (**Figure 6F**). After being able to presurgically locate the tumor, surgery was performed under intraoperative NIRF image guidance (**Figure 6G**).

**Figure 7:**
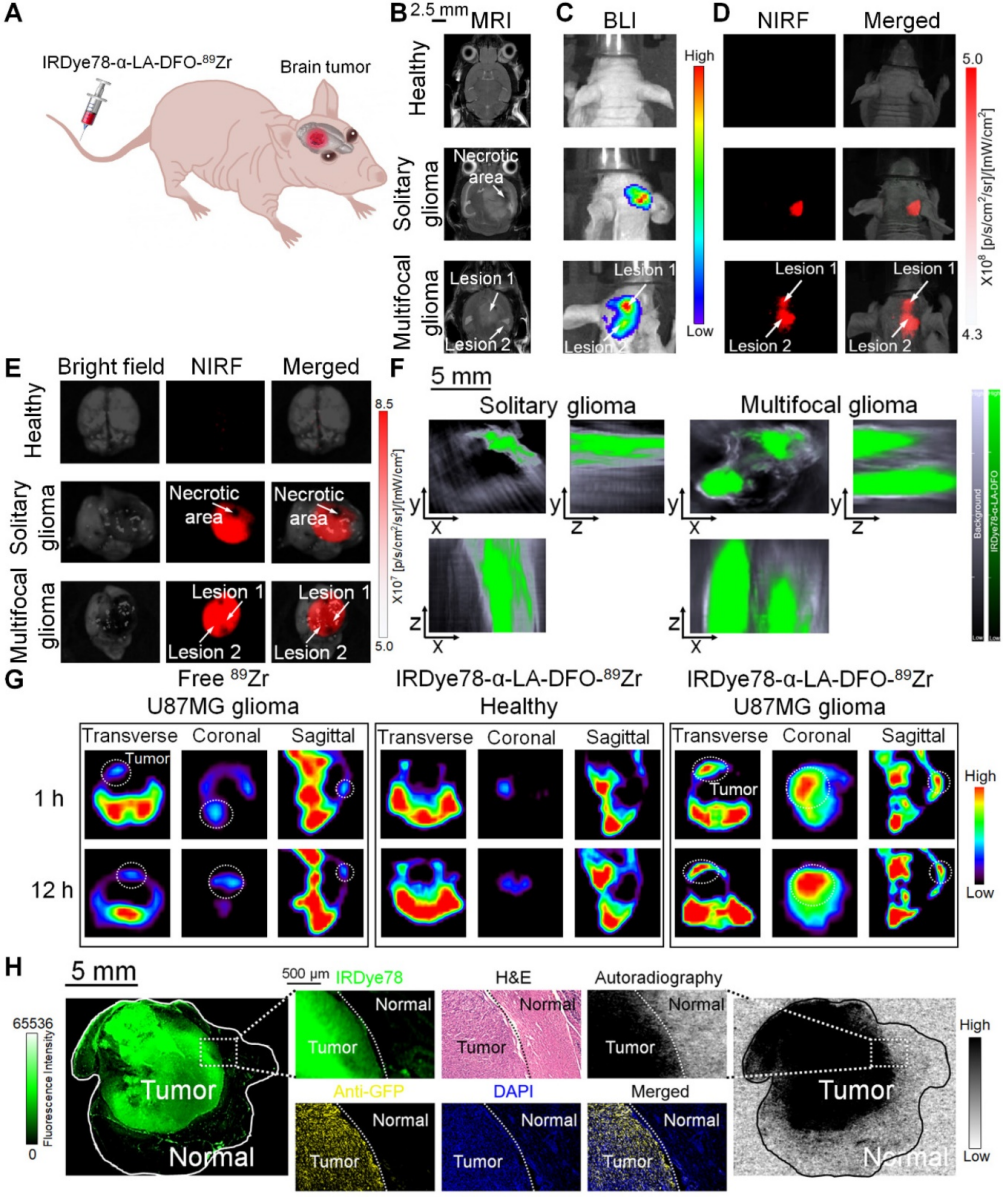
Non-invasive imaging of U-87MG orthotopic brain tumors in living mice with multiple modalities. (A) Schematic view for *in vivo* imaging of a glioblastoma-bearing mouse. (B) Representative 2-dimensional coronal T_2_-weighted MRI images of mice showing a solitary as well as an interconnected bilobed multifocal tumor. (C) Bioluminescence imaging of the solitary and bilobed orthotopic gliomas. (D) Through-skull non-invasive NIR fluorescence imaging of healthy mice and mice with the solitary and bilobed orthotopic gliomas after *i.v.* adminstration of IRDye78-α-LA-DFO. (E) *Ex vivo* fluorescence confirmation of IRDye78-α-LA-DFO uptake in gliomas. (F) MSOT imaging of brains with solitary and bilobed orthotopic gliomas in three dimensions. (G) PET imaging of U-87MG glioma after *i.v.* injection of IRDye78-α-LA-DFO-^89^Zr in contrast to free ^89^Zr and a healthy control without tumors. (H) Histological validation of surgical margins.

In order to extend broader cancer applicability of the IRDye78-α-LA-DFO imaging probe, we employed orthotopic U-87MG glioblastoma mouse models and validated cancerous abnormality by T_2_-weighted MRI and bioluminescence imaging (BLI). In addition to common solitary gliomas, we also observed multifocal gliomas with interconnected macrolobulated lesions (**Figure 7A-C**), closely mimicking human glioma malignancies. Such multifocality, with a 12.8% incidence in humans due to microscopic tumor spread, is associated with poorer prognosis and survival than solitary gliomas 38. IRDye78-α-LA-DFO clearly detected both solitary and bilobed gliomas in mouse models *via* NIRF and MSOT imaging, whereas the healthy control showed negligible uptake into the brain (**Figure 7D-F**). *Ex vivo* fluorescence quantification showed higher signals in gliomas than in healthy brains and contralateral cerebral hemispheres (**Figure S25**). After ^89^Zr-chelation, PET imaging confirmed the higher uptake than free ^89^Zr in U-87MG brain tumors, without crossing the intact blood-brain barrier in healthy controls (**Figure 7G** and **S26**). We histologically examined the distribution of IRDye78-α-LA-DFO-^89^Zr in the brain sections that included the tumor-normal tissue interface.

Dual NIR fluorescence and phosphor autoradiography with intense NIR fluorescence and radioactive signals from IRDye78-α-LA-DFO-^89^Zr confirmed its presence in tumor tissues rather than in adjacent normal tissues. The precise tumor margin correlated well with both H&E and IF staining that identified GFP^+^ U-87MG cells and accurately delineated the actual tumor border as surgical margins with high specificity (**Figure 7H**).

In summary, a single injection of IRDye78-α-LA-DFO-^89^Zr could fulfill the clinical criteria for noninvasive pre- and intraoperative cancer imaging with spatially and temporally complementary modalities, which is otherwise not achievable with current clinical probes. The fluorophore did not suffer from the hydrophobicity, and instability typically associated with ICG for MSOT and against photobleaching. The complex had preferential renal clearance and persistent tumor retention in two representative models of breast and brain cancers. In the case of gliomas, the fluorophore-protein complex enabled through-the-scalp and through-skull imaging of gliomas without craniotomy. The proposed cancer management using the multimodal imaging agent starts with initial preoperative localization of tumors for prudent surgical planning. The long retention and sequestration within the tumor microenvironment then allow intraoperative tumor detection during the surgery days later, from the same injection. Next, NIR fluorescence imaging is scheduled to guide tumor resection, followed by frequent fluorescence histological examination of tumor margins and comparison with conventional H&E staining for dual confirmation. The absence of fluorescence and radioactivity on the resection bed would indicate a successful surgery. Immediate on-site histological validation in the operation room can be feasible using minimized portable systems with multimodalities 39. Completeness of clean margins with preservation of as much functional normal tissues as possible determinatively correlates with improved prognosis, greatly diminishing tumor recurrences, and the need for follow-up secondary resections. In the meantime, although intravenous injection generally leads to much lower immune responses compared to other injection strategies 40, the use of recombinant human α-LA in the process of translation may further decrease the chances of immunogenicity and serve as a prophylactic vaccine autoantigen 41. Further exploration into a broader cancer category using this multimodal agent based on an overlooked but important fluorochrome IRDye78, as well as reduction of multimers to facilitate a higher degree of renal clearance, is expected to unleash its full clinical potential in the near future.

## Supplementary Material

Supplementary figures and tables.

Supplementary video S1.

Supplementary video S2.

Supplementary video S3.

## References

[1] Long NM, Smith CS (2011). Causes and imaging features of false positives and false negatives on F-PET/CT in oncologic imaging. *Insights Imaging*.

[2] Weber J, Beard PC, Bohndiek SE (2016). Contrast agents for molecular photoacoustic imaging. *Nat Methods*.

[3] Shinohara H, Tanaka A, Kitai T, Yanabu N, Inomoto T, Satoh S (1996). Direct measurement of hepatic indocyanine green clearance with near-infrared spectroscopy: separate evaluation of uptake and removal. *Hepatology*.

[4] Napp J, Stammes MA, Claussen J, Prevoo HAJM, Sier CFM, Hoeben FJM (2018). Fluorescence- and multispectral optoacoustic imaging for an optimized detection of deeply located tumors in an orthotopic mouse model of pancreatic carcinoma. *Int J Cancer*.

[5] Attia ABE, Ho CJH, Chandrasekharan P, Balasundaram G, Tay HC, Burton NC (2016). Multispectral optoacoustic and MRI coregistration for molecular imaging of orthotopic model of human glioblastoma. *J Biophotonics*.

[6] Dogra V, Chinni B, Singh S, Schmitthenner H, Rao N, Krolewski JJ (2016). Photoacoustic imaging with an acoustic lens detects prostate cancer cells labeled with PSMA-targeting near-infrared dye-conjugates. *J Biomed Opt*.

[7] Singha S, Kim D, Roy B, Sambasivan S, Moon H, Rao AS (2015). A structural remedy toward bright dipolar fluorophores in aqueous media. *Chem Sci*.

[8] Choi HS, Nasr K, Alyabyev S, Feith D, Lee JH, Kim SH (2011). Synthesis and *in vivo* fate of zwitterionic near-infrared fluorophores. *Angew Chem Int Ed Engl*.

[9] Nakayama A, del Monte F, Hajjar RJ, Frangioni JV (2002). Functional near-infrared fluorescence imaging for cardiac surgery and targeted gene therapy. *Mol Imaging*.

[10] Zaheer A, Lenkinski RE, Mahmood A, Jones AG, Cantley LC, Frangioni JV (2001). *In vivo* near-infrared fluorescence imaging of osteoblastic activity. *Nat Biotechnol*.

[11] Xin J, Zhang X, Liang J, Xia L, Yin J, Nie Y (2013). *In vivo* gastric cancer targeting and imaging using novel symmetric cyanine dye-conjugated GX1 peptide probes. *Bioconjug Chem*.

[12] Commisso C, Davidson SM, Soydaner-Azeloglu RG, Parker SJ, Kamphorst JJ, Hackett S (2013). Macropinocytosis of protein is an amino acid supply route in Ras-transformed cells. *Nature*.

[13] Palm W, Park Y, Wright K, Pavlova NN, Tuveson DA, Thompson CB (2015). The utilization of extracellular proteins as nutrients is suppressed by mTORC1. *Cell*.

[14] Gao FP, Lin YX, Li LL, Liu Y, Mayerhoffer U, Spenst P (2014). Supramolecular adducts of squaraine and protein for noninvasive tumor imaging and photothermal therapy *in vivo*. *Biomaterials*.

[15] Chen Q, Wang C, Zhan Z, He W, Cheng Z, Li Y (2014). Near-infrared dye bound albumin with separated imaging and therapy wavelength channels for imaging-guided photothermal therapy. *Biomaterials*.

[16] Antaris AL, Chen H, Diao S, Ma Z, Zhang Z, Zhu S (2017). A high quantum yield molecule-protein complex fluorophore for near-infrared II imaging. *Nat Commun*.

[17] Zhao H, Chao Y, Liu J, Huang J, Pan J, Guo W (2016). Polydopamine coated single-walled carbon nanotubes as a versatile platform with radionuclide labeling for multimodal tumor imaging and therapy. *Theranostics*.

[18] Liu Y, Ashton JR, Moding EJ, Yuan H, Register JK, Fales AM (2015). A plasmonic gold nanostar theranostic probe for *in vivo* tumor imaging and photothermal therapy. *Theranostics*.

[19] Wang J, Liu L, You Q, Song Y, Sun Q, Wang Y (2018). All-in-one theranostic nanoplatform based on hollow MoS_x_ for photothermally-maneuvered oxygen self-enriched photodynamic therapy. *Theranostics*.

[20] Yang L, Wang J, Yang S, Lu Q, Li P, Li N (2019). Rod-shape MSN@MoS_2_ nanoplatform for FL/MSOT/CT imaging-guided photothermal and photodynamic therapy. *Theranostics*.

[21] Yang J, Dai D, Lou X, Ma L, Wang B, Yang Y-W (2020). Supramolecular nanomaterials based on hollow mesoporous drug carriers and macrocycle-capped CuS nanogates for synergistic chemo-photothermal therapy. *Theranostics*.

[22] Wu K, Zhao H, Sun Z, Wang B, Tang X, Dai Y (2019). Endogenous oxygen generating multifunctional theranostic nanoplatform for enhanced photodynamic-photothermal therapy and multimodal imaging. *Theranostics*.

[23] Wang Y, Zhang W, Sun P, Cai Y, Xu W, Fan Q (2019). A novel multimodal NIR-II nanoprobe for the detection of metastatic lymph nodes and targeting chemo-photothermal therapy in oral squamous cell carcinoma. *Theranostics*.

[24] Hu X, Tang Y, Hu Y, Lu F, Lu X, Wang Y (2019). Gadolinium-chelated conjugated polymer-based nanotheranostics for photoacoustic/magnetic resonance/NIR-II fluorescence imaging-guided cancer photothermal therapy. *Theranostics*.

[25] Komljenovic D, Wiessler M, Waldeck W, Ehemann V, Pipkorn R, Schrenk H-H (2016). NIR-cyanine dye linker: a promising candidate for isochronic fluorescence imaging in molecular cancer diagnostics and therapy monitoring. *Theranostics*.

[26] Lütje S, Heskamp S, Franssen GM, Frielink C, Kip A, Hekman M (2019). Development and characterization of a theranostic multimodal anti-PSMA targeting agent for imaging, surgical guidance, and targeted photodynamic therapy of PSMA-expressing tumors. *Theranostics*.

[27] Benson RC, Kues HA (1978). Fluorescence properties of indocyanine green as related to angiography. *Phys Med Biol*.

[28] Zeglis BM, Lewis JS (2015). The bioconjugation and radiosynthesis of ^89^Zr-DFO-labeled antibodies. *J Vis Exp*.

[29] Jacobsen MT, Fairhead M, Fogelstrand P, Howarth M (2017). Amine landscaping to maximize protein-dye fluorescence and ultrastable protein-ligand interaction. *Cell Chem Biol*.

[30] Dilworth JR, Pascu SI (2018). The chemistry of PET imaging with zirconium-89. *Chem Soc Rev*.

[31] Moroz MA, Huang R, Kochetkov T, Shi W, Thaler H, de Stanchina E (2011). Comparison of corticotropin-releasing factor, dexamethasone, and temozolomide: treatment efficacy and toxicity in U87 and C6 intracranial gliomas. *Clin Cancer Res*.

[32] Jiao L, Song F, Cui J, Peng X (2018). A near-infrared heptamethine aminocyanine dye with a long-lived excited triplet state for photodynamic therapy. *Chem Commun*.

[33] Zhang Z, Kao J, D'Avignon A, Achilefu S (2010). Understanding dichromic fluorescence manifested in certain ICG analogs. *Pure Appl Chem*.

[34] Kraft JC, Ho RJ (2014). Interactions of indocyanine green and lipid in enhancing near-infrared fluorescence properties: the basis for near-infrared imaging *in vivo*. *Biochemistry*.

[35] Ruggiero A, Villa CH, Bander E, Rey DA, Bergkvist M, Batt CA (2010). Paradoxical glomerular filtration of carbon nanotubes. *Proc Natl Acad Sci U S A*.

[36] Klohs J, Wunder A, Licha K (2008). Near-infrared fluorescent probes for imaging vascular pathophysiology. *Basic Res Cardiol*.

[37] Liu J, Yu M, Zhou C, Yang S, Ning X, Zheng J (2013). Passive tumor targeting of renal-clearable luminescent gold nanoparticles: long tumor retention and fast normal tissue clearance. *J Am Chem Soc*.

[38] Patil CG, Yi A, Elramsisy A, Hu J, Mukherjee D, Irvin DK (2012). Prognosis of patients with multifocal glioblastoma: a case-control study. *J Neurosurg*.

[39] Shen B, Yan J, Wang S, Zhou F, Zhao Y, Hu R (2020). Label-free whole-colony imaging and metabolic analysis of metastatic pancreatic cancer by an autoregulating flexible optical system. *Theranostics*.

[40] Zhang L, Wang W, Wang S (2015). Effect of vaccine administration modality on immunogenicity and efficacy. *Expert Rev Vaccines*.

[41] Jaini R, Kesaraju P, Johnson JM, Altuntas CZ, Jane-Wit D, Tuohy VK (2010). An autoimmune-mediated strategy for prophylactic breast cancer vaccination. *Nat Med*.

[42] Tien MZ, Meyer AG, Sydykova DK, Spielman SJ, Wilke CO (2013). Maximum allowed solvent accessibilities of residues in proteins. *PloS One*.

