# Supplementary material for: Structurally symmetric near-infrared fluorophore IRDye78-protein complex enables multimodal cancer imaging
Source: Theranostics. 2021 Jan 1;11(6):2534–49. doi: 10.7150/thno.54928 (PMC7806473; doi:10.7150/thno.54928)
Supplement: Supplementary file 1 — Supplementary figures and tables. [file thnov11p2534s1.pdf]

# Supporting Information

**Supplementary Tables**

**Table S1.** High-resolution mass spectroscopy (HRMS) of Compound **3** in amu units, including analysis of multiply charged species which result from negatively charged sulfonates.

| **Compound number name** | **Calculated m-1** | **Calculated [m-2]/2** | **Calculated [m-3]/3** |
|---|---|---|---|
| (3) Chloro dye precursor to IRDye78 | 885.1620966 | 442.0770783 | 294.3820722 |
| **Formula** | **Found m-1** | **Found [m-2]/2** | **Found [m-3]/3** |
| $C_{38}H_{47}C_lN_2O_{12}S_4$ | 885.1608 | N/A | 294.3814 |
| **Exact mass** | **m-1 diff** | **[m-2]/2 diff** | **[m-3]/3 diff** |
| 886.1700366 | 0.00129664 | N/A | 0.000672214 |
| | **m-1 ppm** | **[m-2]/2 ppm** | **[m-3]/3 ppm** |
| | 1.465 E-06 | none | 2.2835 E-06 |

**Table S2.** HRMS characterization of Compound **4** in amu units, including analysis of multiply charged species which result from negatively charged sulfonates.

| **Compound number name** | **Calculated m-1** | **Calculated [m-2]/2** | **Calculated [m-3]/3** |
|---|---|---|---|
| (4) IRDye78 | 1016.25635 | 507.12035 | 337.7442917 |
| **Formula** | **Found m-1** | **Found [m-2]/2** | **Found [m-3]/3** |
| $C_{47}H_{56}N_2O_{15}S_4$ | 1016.2496 | 507.1202 | 337.7436 |
| **Exact mass** | **m-1 diff** | **[m-2]/2 diff** | **[m-3]/3 diff** |
| 1017.26418 | 0.00675 | 0.00015 | 0.0006912 |
| | **m-1 ppm** | **[m-2]/2 ppm** | **[m-3]/3 ppm** |
| | 6.642 E-06 | 2.957 E-07 | 2.048 E-06 |

**Supplementary Figures**

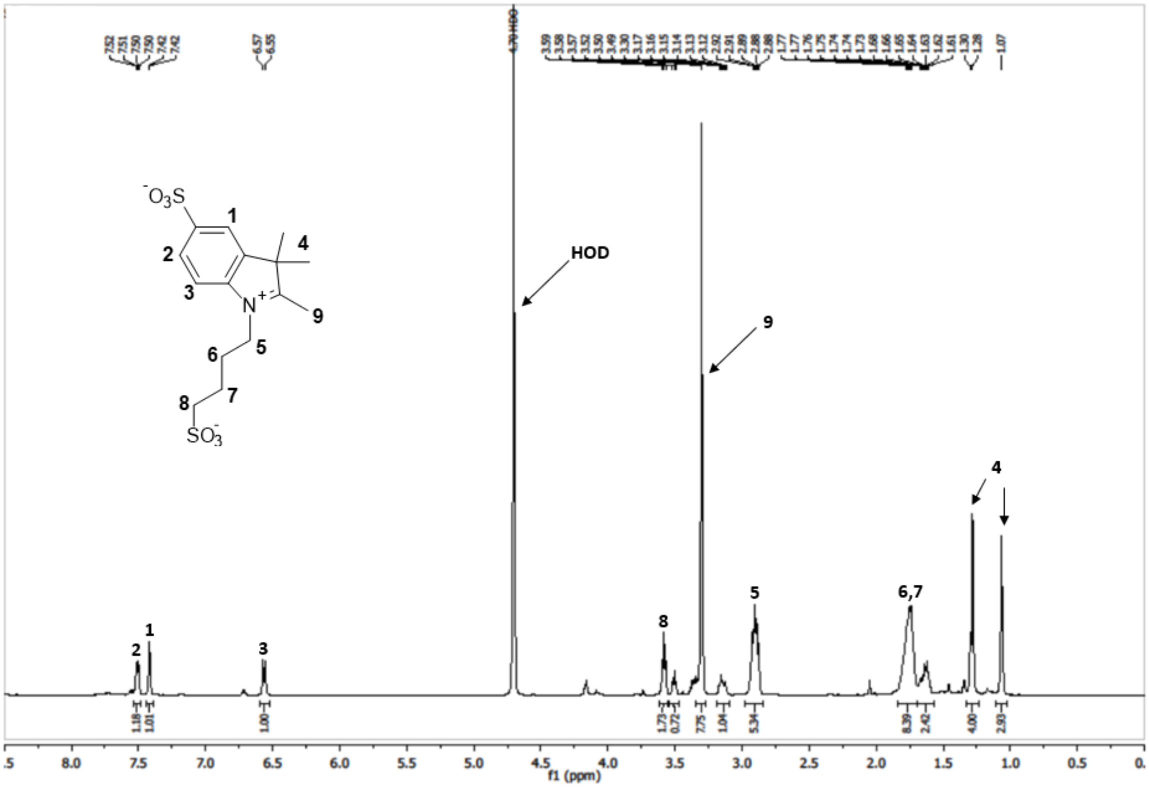


**Figure S1.** $^1$H NMR spectrum ($D_2O$, 500 MHz) of 2,3,3-trimethyl-1-(4-sulfonatobutyl)-3H-indol-1-ium-5-sulfonate (**2**).

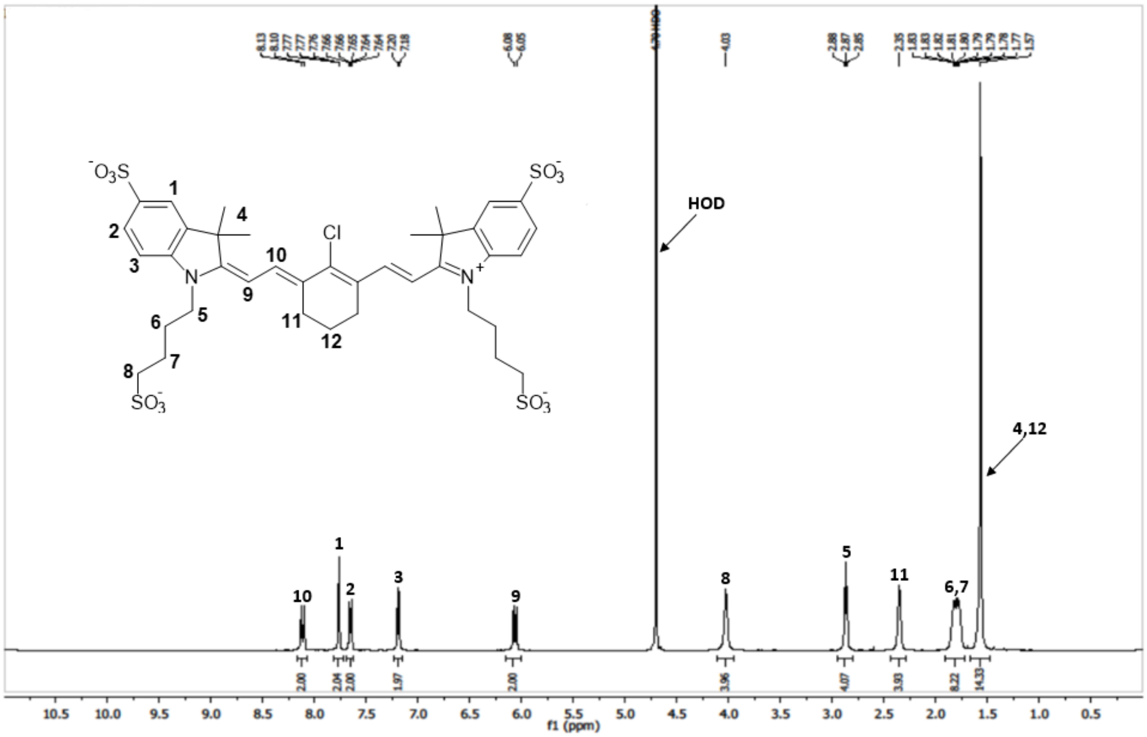


**Figure S2.** $^1$H NMR spectrum ($D_2O$, 500 MHz) of 2-((E)-2-((E)-2-chloro-3-(2-((E)-3,3-dimethyl-5-sulfonato-1-(4-sulfonatobutyl)indolin-2-ylidene) ethylidene)cyclohex-1-en-1-yl)vinyl)-3,3-dimethyl-1-(4-sulfonatobutyl)-3H-indol-1-ium-5-sulfonate (**4**).

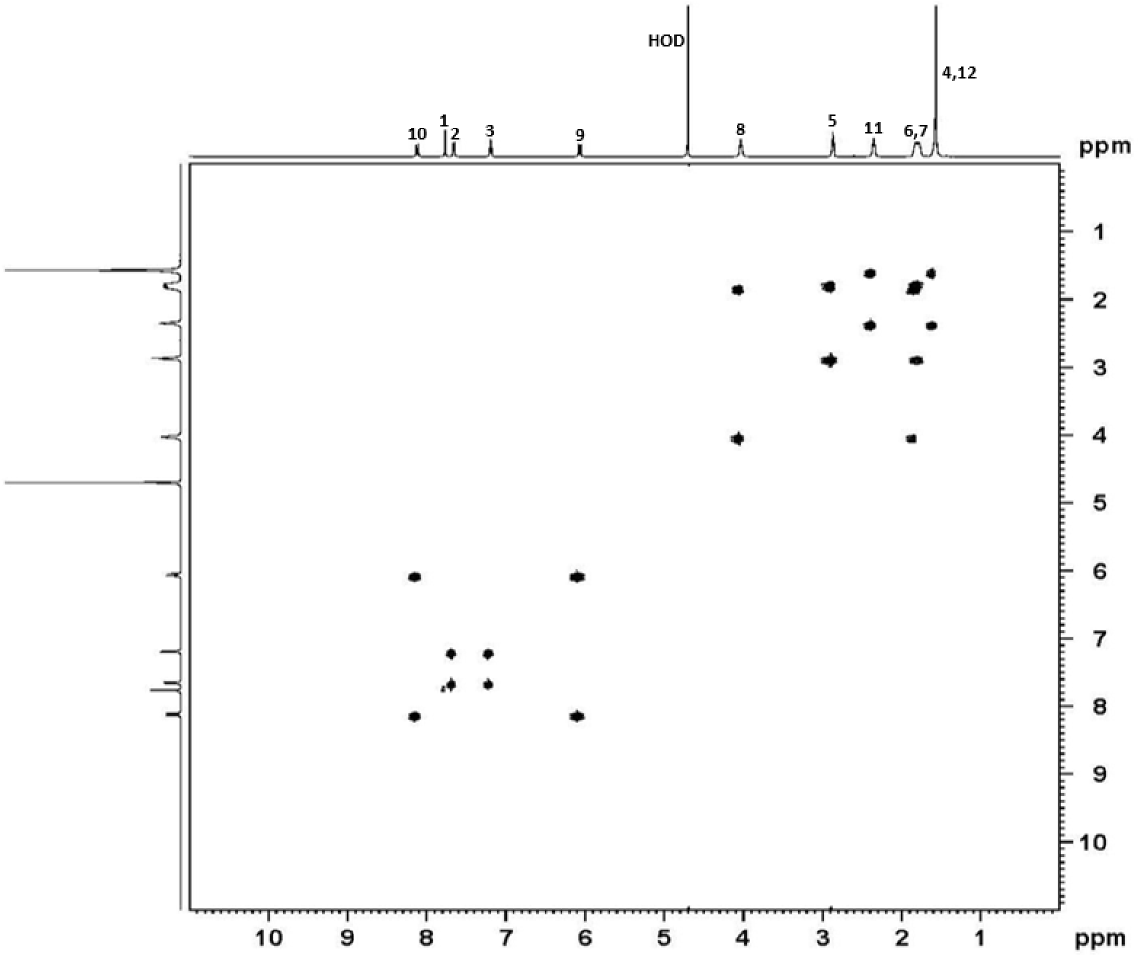


**Figure S3.** $^1H$-$^1H$ DQF-COSY NMR spectrum of 2-((E)-2-((E)-2-chloro-3-(2-((E)-3,3-dimethyl-5-sulfonato-1-(4-sulfonatobutyl)indolin-2-ylidene)ethylidene)cyclohex-1-en-1-yl)vinyl)-3,3-dimethyl-1-(4-sulfonatobutyl)-3H-indol-1-ium-5-sulfonate (**4**).

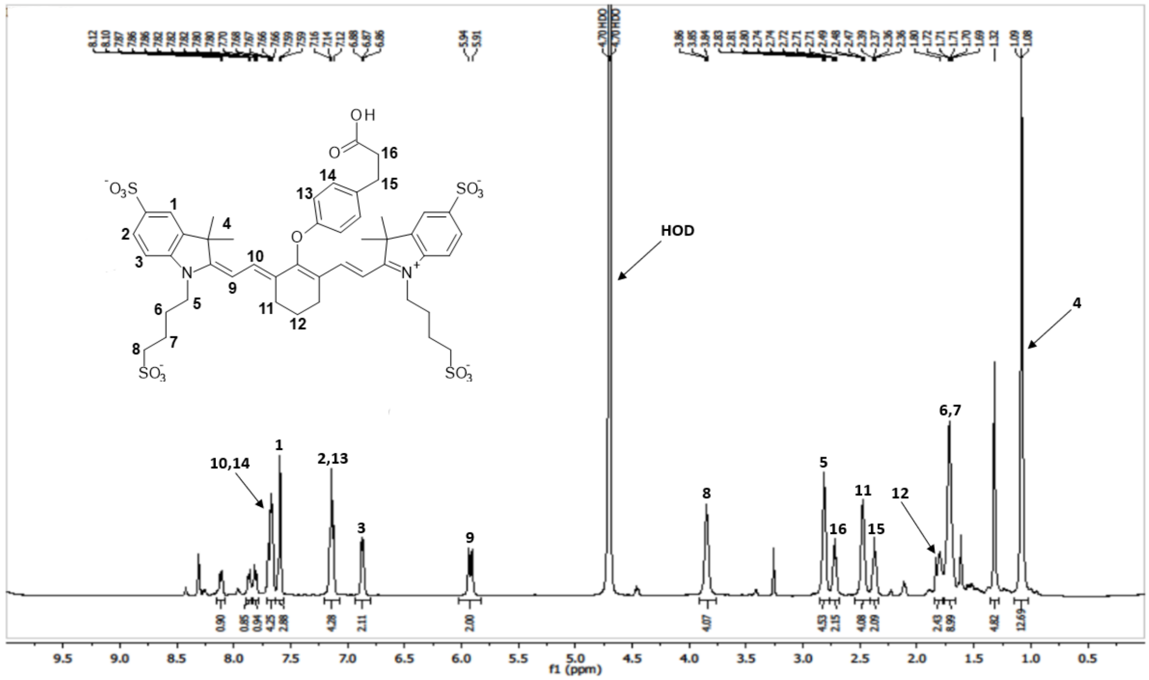


**Figure S4.** $^{1}$H NMR spectrum ($D_2O$, 500 MHz) of (2-((E)-2-((E)-2-(4-(2-carboxyethyl)phenoxy)-3-(2-((E)-3,3-dimethyl-5-sulfonato-1-(4-sulfonatobutyl)indolin-2-ylidene)ethylidene)cyclohex-1-en-1-yl)vinyl)-3,3-dimethyl-1-(4-sulfonatobutyl)-3H-indol-1-ium-5-sulfonate (**5**).

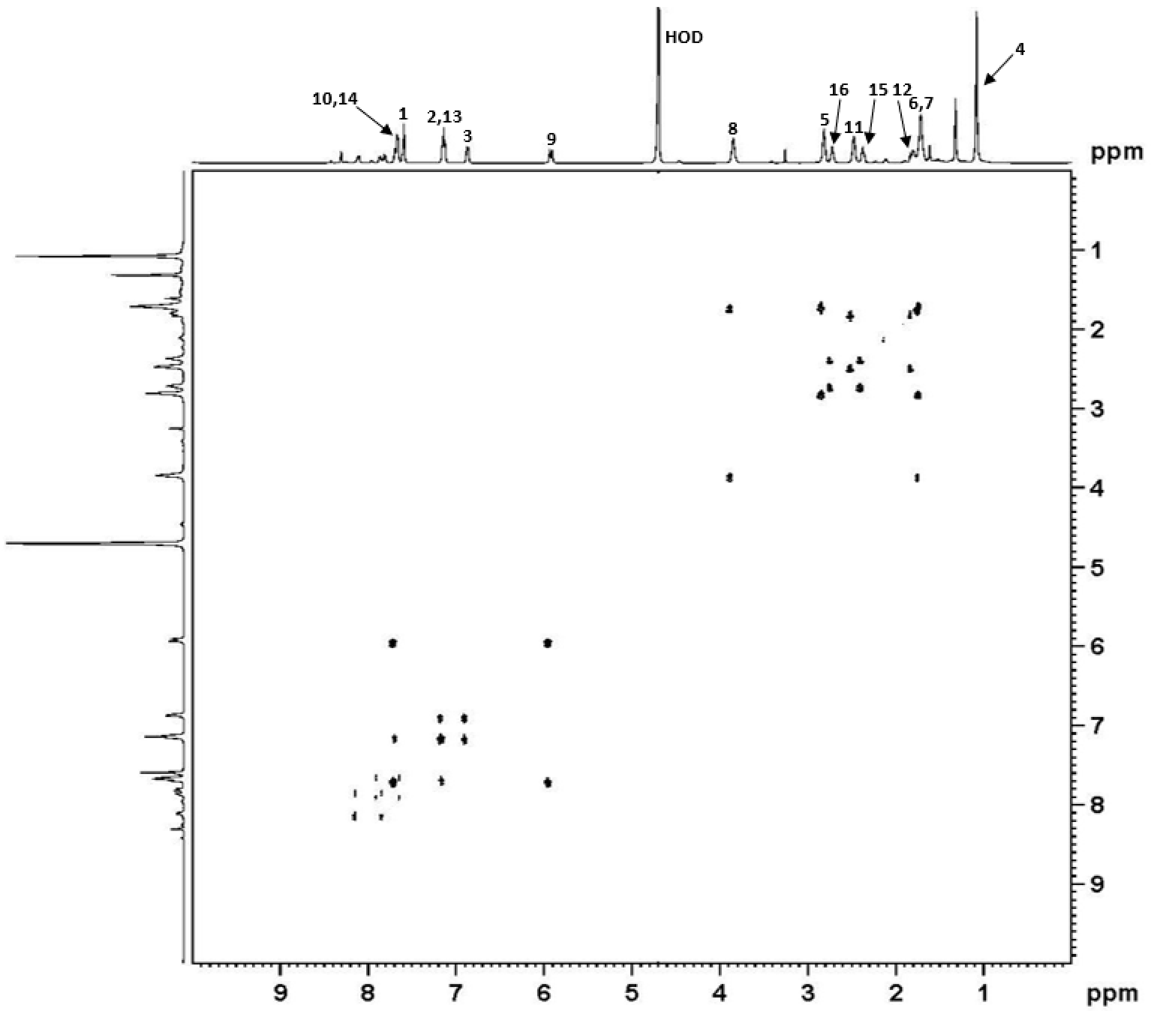


**Figure S5.** $^{1}H$-$^{1}H$ DQF-COSY NMR spectrum of (2-((E)-2-((E)-2-(4-(2-carboxyethyl)phenoxy)-3-(2-((E)-3,3-dimethyl-5-sulfonato-1-(4-sulfonatobutyl)indolin-2-ylidene)ethylidene)cyclohex-1-en-1-yl)vinyl)-3,3-dimethyl-1-(4-sulfonatobutyl)-3H-indol-1-ium-5-sulfonate (**5**).

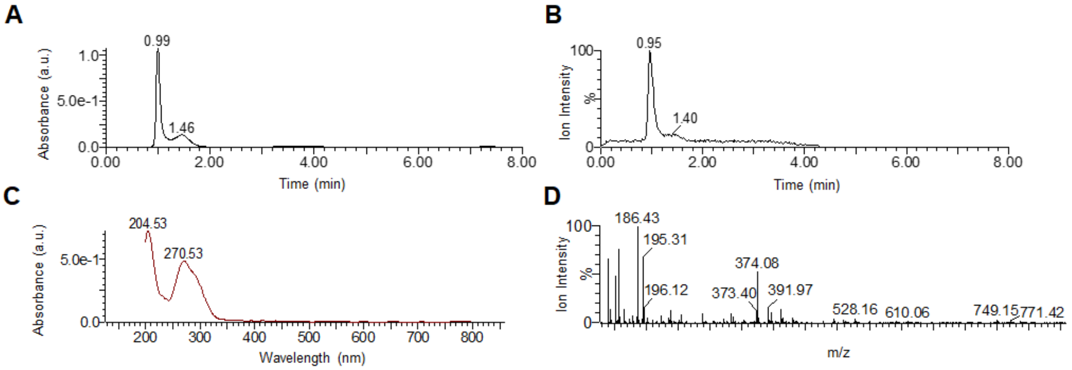


**Figure S6.** LC-MS characterization of 2,3,3-trimethyl-1-(4-sulfonatobutyl)-3H-indol-1-ium-5-sulfonate (**2**) with a 10-100% methanol-aqueous gradient. (a) 270 nm single-wavelength chromatogram. (b) $ES^-$ total ion chromatogram (TIC). (c) Diode array spectrum. (d) Electrospray ($ES^-$) mass spectrum.

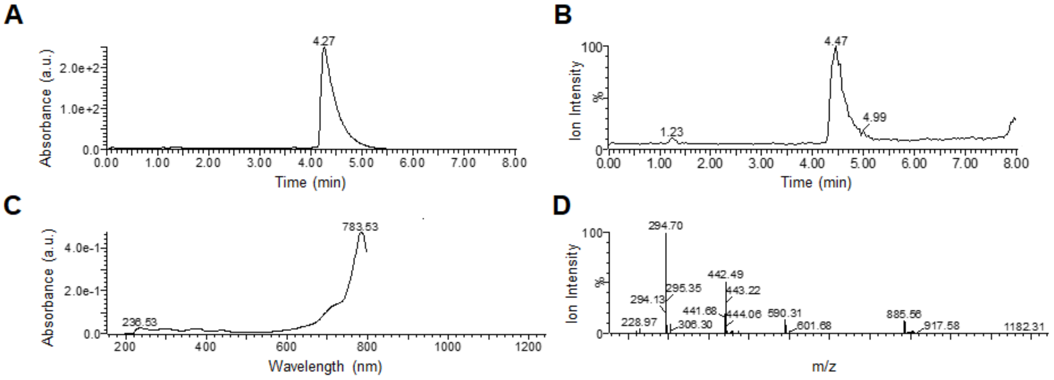


**Figure S7.** LC-MS characterization of 2-((E)-2-((E)-2-chloro-3-(2-((E)-3,3-dimethyl-5-sulfonato-1-(4-sulfonatobutyl)indolin-2-ylidene) ethylidene)cyclohex-1-en-1-yl)vinyl)-3,3-dimethyl-1-(4-sulfonatobutyl)-3H-indol-1-ium-5-sulfonate (**4**) with a 20-80% methanol-aqueous gradient. (a) Total diode array (TDA) chromatogram. (b) $ES^-$ TIC. (c) Diode array spectrum. (d) Electrospray ($ES^-$) mass spectrum.

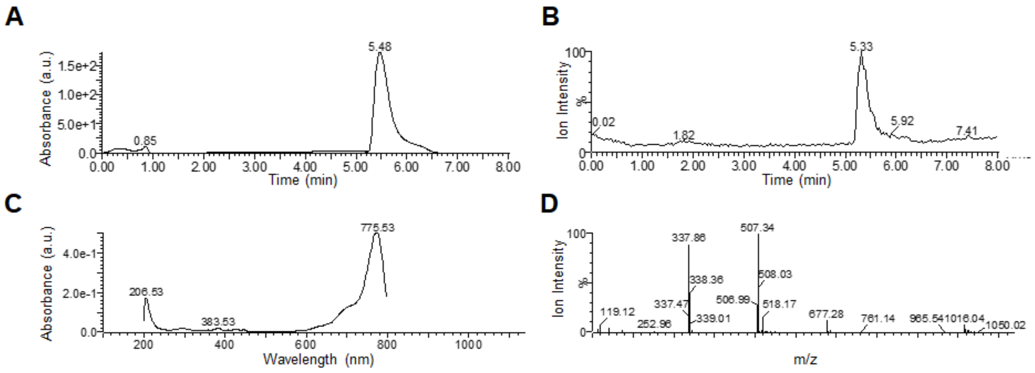


**Figure S8.** LC-MS characterization of (2-((E)-2-((E)-2-(4-(2-carboxyethyl)phenoxy)-3-(2-((E)-3,3-dimethyl-5-sulfonato-1-(4-sulfonatobutyl)indolin-2-ylidene)ethylidene)cyclohex-1-en-1-yl)vinyl)-3,3-dimethyl-1-(4-sdaulfonatobutyl)-3H-indol-1-ium-5-sulfonate (**5**) with a 20-80% methanol-aqueous gradient. (a) TDA chromatogram. (b) $ES^-$ TIC. (c) Diode array spectrum. (d) Electrospray ($ES^-$) mass spectrum.

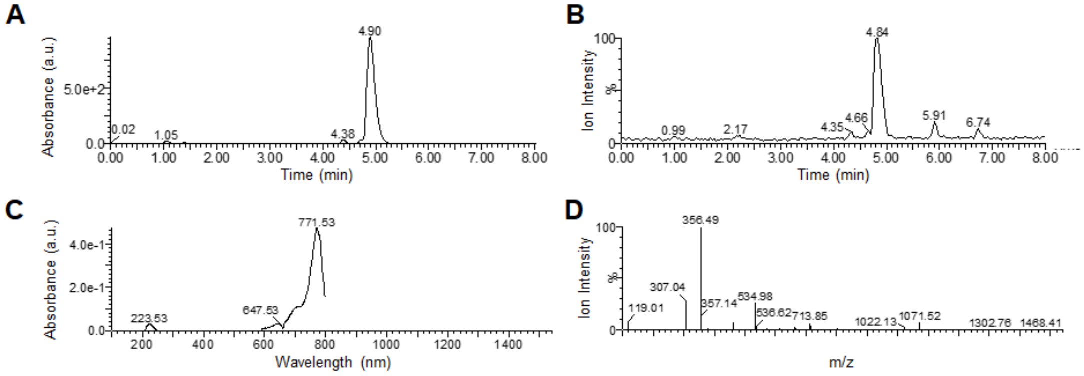


**Figure S9.** LC-MS characterization of butyl amine quenched 2-((E)-2-((E)-3-(2-((E)-3,3-dimethyl-5-sulfonato-1-(4-sulfonatobutyl)indolin-2-ylidene)ethylidene)-2-(4-(3-((2,5-dioxopyrrolidin-1-yl)oxy)-3-oxopropyl)phenoxy)cyclohex-1-en-1-yl)vinyl)-3,3-dimethyl-1-(4-sulfonatobutyl)-3H-indol-1-ium-5-sulfonate (**6**). A trace analytical amount (0.1-0.2 mg) was dissolved in 0.1% butyl amine in water with a 20-80% methanol-aqueous gradient. (a) TDA chromatogram. (b) $ES^-$ TIC. (c) Diode array spectrum. (d) Electrospray ($ES^-$) mass spectrum.

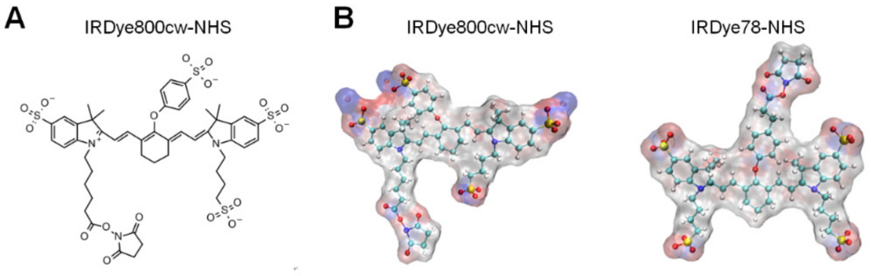


**Figure S10.** (a) Chemical structure of asymmetric IRDye800cw NHS ester. (b) Merged visualization of molecular structures and ESP charge distribution (Figure 1E and 1F) for IRDye800cw and IRDye78 NHS esters, showing their partially asymmetric and symmetric ESP distributions, respectively.

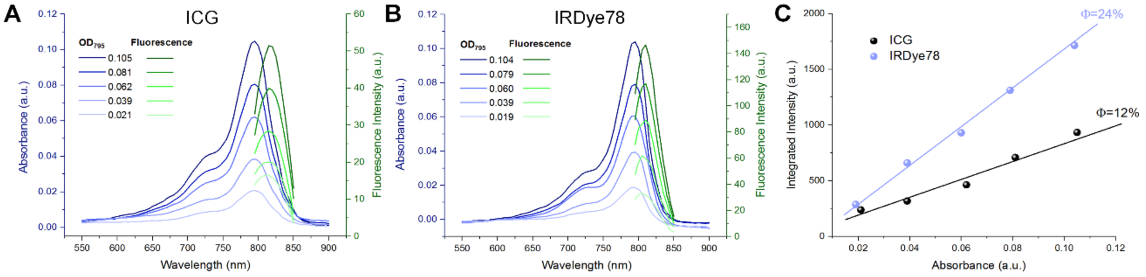


**Figure S11.** QY measurements of (a) ICG and (b) IRDye78. UV-vis absorption and fluorescence corresponding to different optical densities of each spectrum at 795 nm were measured in DMSO. (c) A plot of integrated fluorescence as a function of absorbance for IRDye78 and the fluorescence standard ICG to derive QY from slopes of linear fits.

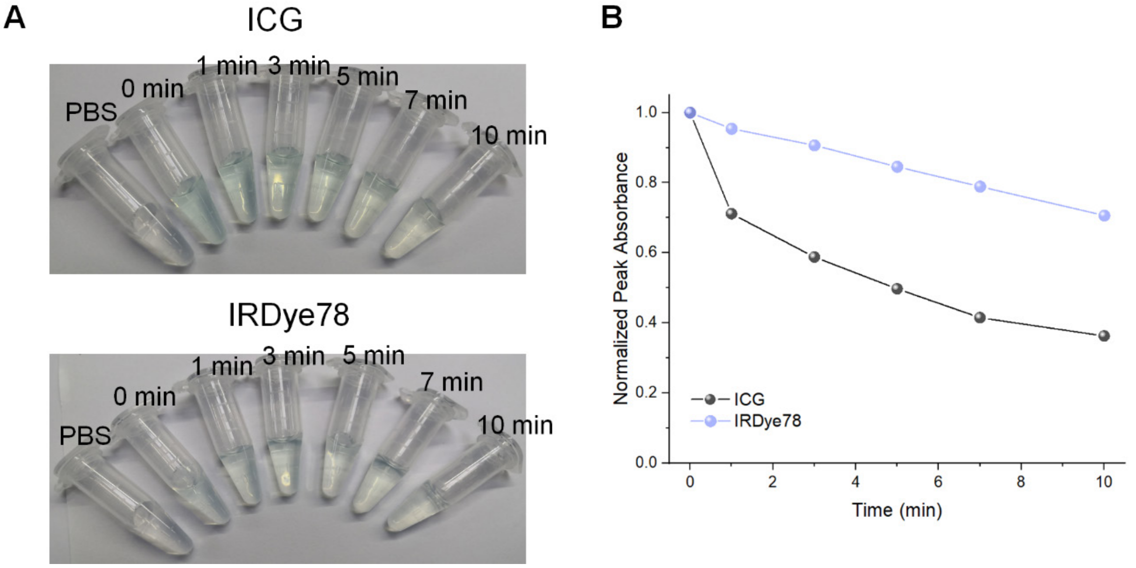


**Figure S12.** Photobleaching of ICG and IRDye78. (a) Photographs showed a more obvious dye fading of ICG than IRDye78 upon different irradiation time with a NIR laser (808 nm, Daheng Science and Technology) at a power density of 0.1 W/cm$^2$. (b) Peak absorbance further evidenced a more drastic decrease in ICG, indicating IRDye78 has a superior photostability against photobleaching than ICG.

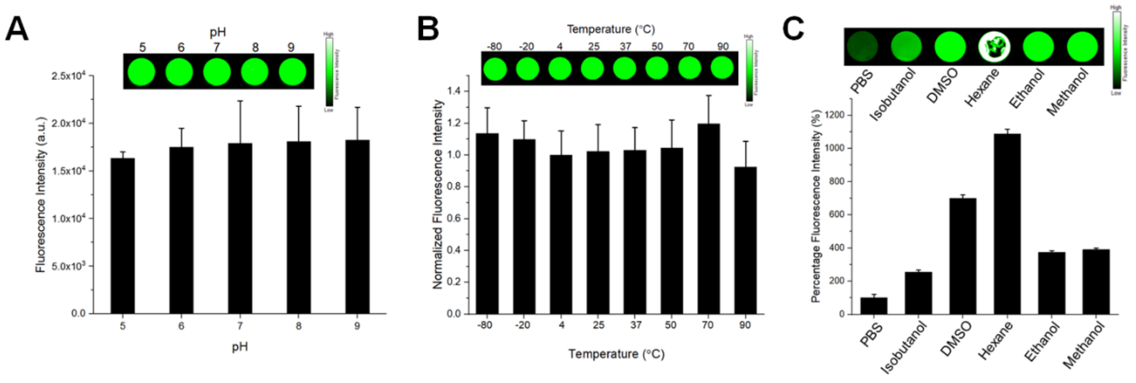


**Figure S13.** Fluorescence intensities of IRDye78 (a) at physiological pH ranging from 5 to 9 in PBS, (b) after 2 h incubation at different temperatures from -80 to 90 ºC, and (c) in different organic solvents. Insets show the corresponding NIR fluorescence images (c is rescaled for better observation of signal differences).

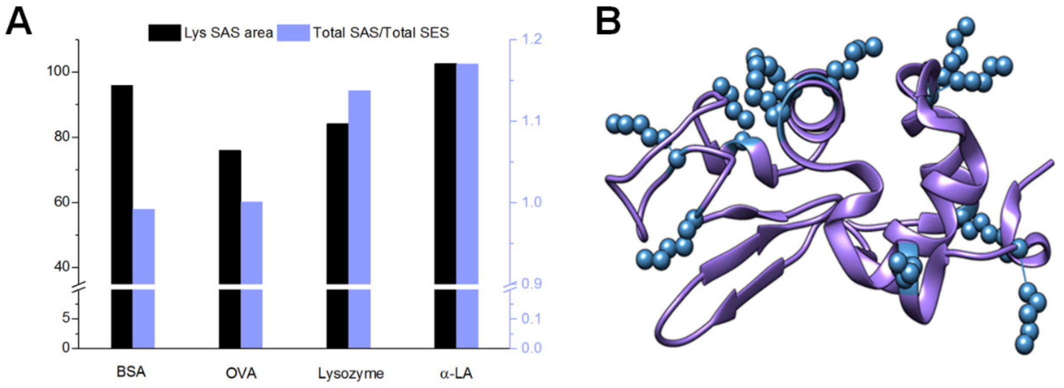


**Figure S14.** (a) Comparison of Lys solvent accessible surface (SAS) area (black) and the ratio of total solvent accessible and total solvent exclusive surface (SES) areas (blue) of all amino acid residues in monomeric α-LA (PDB code: 1F6R) and common carrier proteins including bovine serum albumin (BSA, PDB code: 4F5S), ovalbumin (OVA, PDB code: 1OVA) and lysozyme (PDB code:1DPX). α-LA has the largest Lys SAS area and total SAS/SES ratio among all. BSA is generally considered to have approximately 35 sterically available primary amines out of a total of 59 (59.3%), which are reactive for chemical modification from Lys side chains corresponding to a cutoff SAS area=87.54 Å$^2$. The percentages of reactive Lys in OVA, lysozyme and α-LA are 30.0% (6/20), 66.7% (4/6) and 66.7% (8/12) respectively. Noticeably, α-LA with a much smaller molecular weight even has more reactive Lys residues than OVA. (b) Ball-stick illustration of Lys positions (cyan) in the α-LA structure.

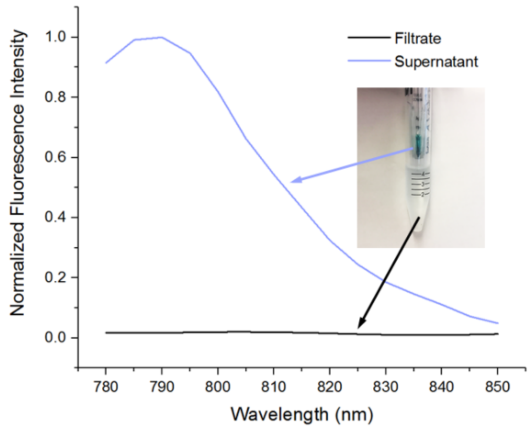


**Figure S15.** Fluorescence spectra of filtrate and supernatant collected from a 3 kDa cut-off molecular weight filter after ultracentrifugation. Dominant fluorescence was observed in the retained supernatant in contrast to negligibly low fluorescence in the filtrate, showing a high chemical conjugation efficiency, thanks to the multiple reactive surface Lys residues of α-LA and the high purity of NHS reactive groups (>95% as assayed in the final synthesis step) on IRDye78.

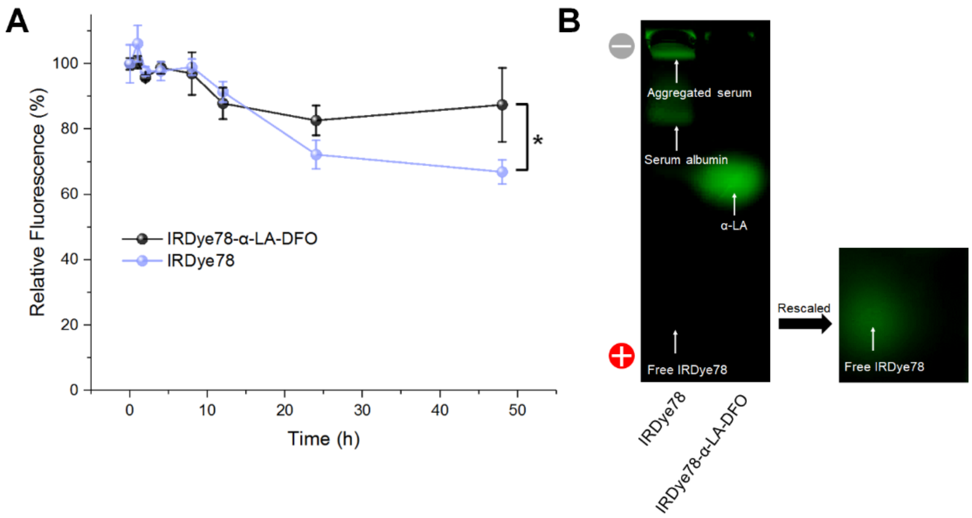


**Figure S16.** Serum interactions of IRDye78 and IRDye78-α-LA-DFO. (a) NIR fluorescence intensities of IRDye78 small molecule dye and IRDye78-α-LA-DFO complex as a function of incubation time at 37 °C in 50% mouse serum. After 48 h, the changes in fluorescence intensity became statistically significant ($P<0.05$). (b) Agarose gel electrophoresis of equimolar IRDye78 and IRDye78-α-LA-DFO after 24 h incubation in 50% mouse serum. IRDye78 strongly interacts with serum proteins with the majority interacting with aggregates and serum albumin, and a small fraction still in the form of small molecules (as rescaled for better visualization). The IRDye78-α-LA-DFO, however, does not interact with serum as observed by the band at a smaller molecular weight.

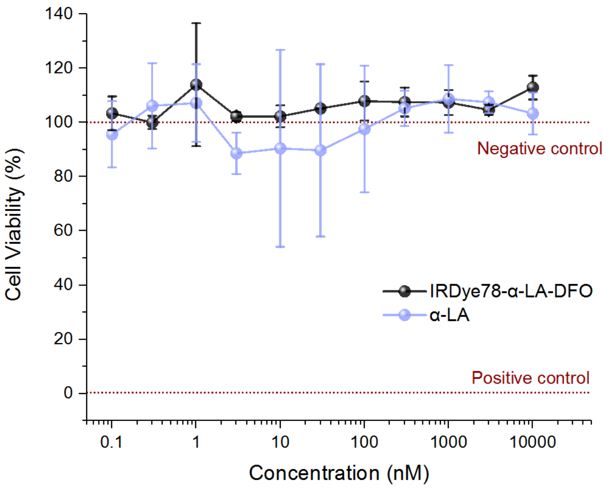


**Figure S17.** Investigation of in vitro cytotoxicity of IRDye78-α-LA-DFO in MDA-MB-231 cancer cells at various concentrations with native α-LA as a control using WST-8 assays. 1 mM SDS was employed as the positive control.

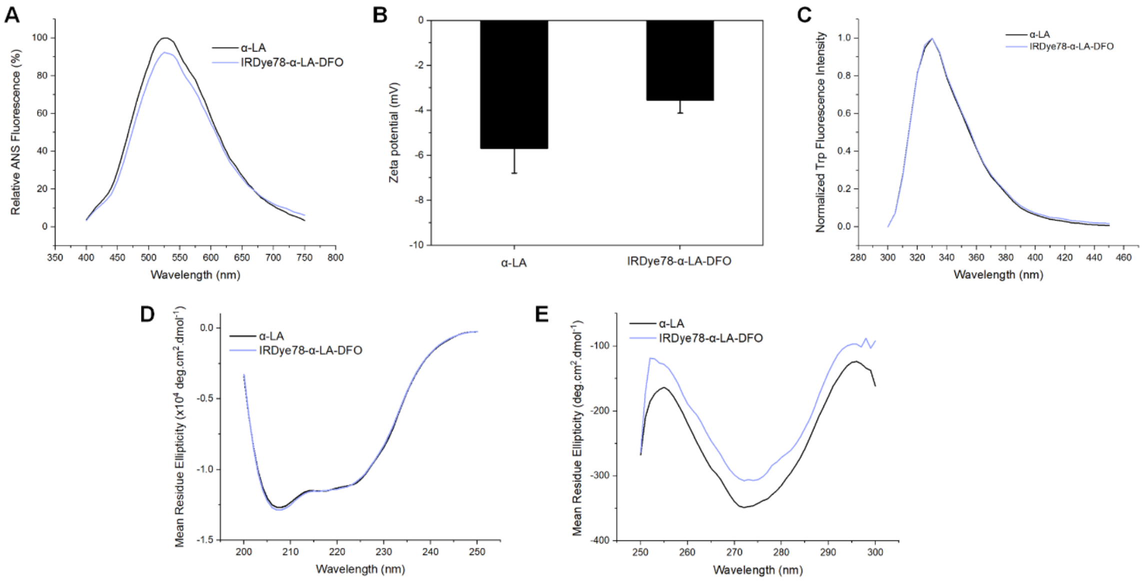


**Figure S18.** Characterization of IRDye78-α-LA-DFO. (a) ANS fluorescence, (b) zeta potentials, (c) intrinsic tryptophan fluorescence spectra, (d) far-ultraviolet (UV) and (e) near-UV CD spectra of α-LA and IRDye78-α-LA-DFO. ANS fluorescence decreased after chemical conjugation of IRDye78 and DFO representing increased hydrophilicity, while the zeta potential insignificantly dropped but was still kept at a low value of <-10 mV due to the stoichiometry we used, with more molarity of positively-charged DFO that buffers the negative charge of the dye resulting in a well-balanced zeta potential. The peak of intrinsic tryptophan fluorescence emission stayed unshifted without distinct conformational changes in the protein structure after covalent modification. Furthermore, the far-UV CD spectra did not show any major variations in all rigid secondary structures after covalent modification. Similarly, the characteristics of near-UV CD spectra also remained almost identical before and after modification with only a minor decrease in CD signals within error limits. Therefore, the tertiary structure is also largely maintained.

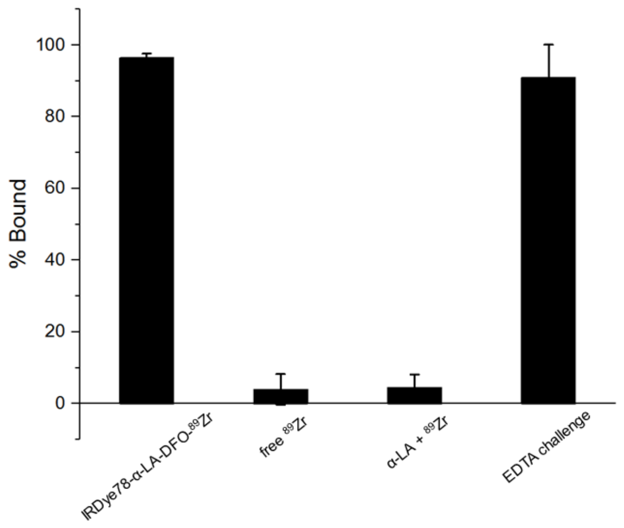


**Figure S19.** Percent $^{89}Zr$ radioisotope bound calculated from iTLC for IRDye78-α-LA-DFO-$^{89}Zr$, free $^{89}Zr$, α-LA with $^{89}Zr$, and IRDye78-α-LA-DFO-$^{89}Zr$ subject to 2 h EDTA challenge at room temperature.

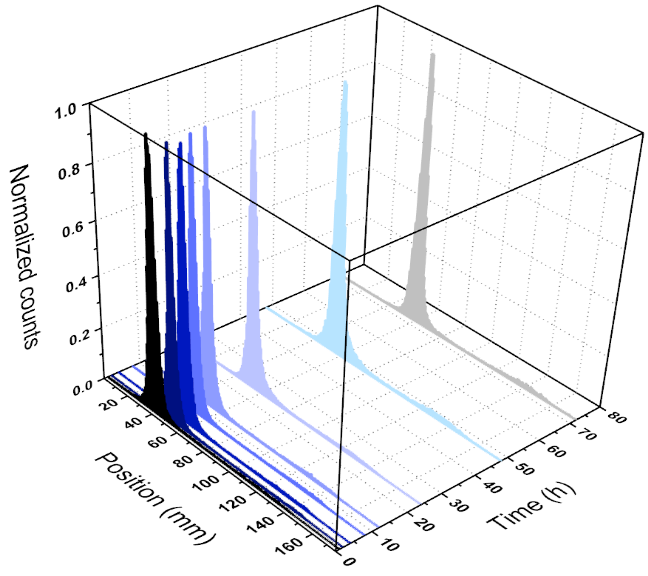


**Figure S20.** Instant thin-layer chromatographs of radiolabeled IRDye78-α-LA-DFO-$^{89}Zr$ over 72 h time periods at 37 °C in mouse serum.

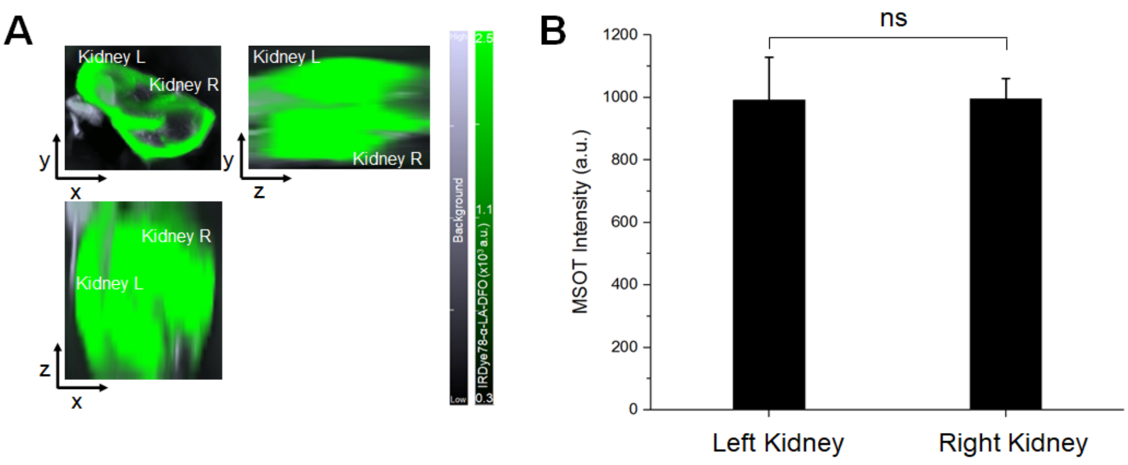


**Figure S21.** (a) Representative ex vivo 3D MSOT images of kidneys 1 h *p.i.* of IRDye78-α-LA-DFO. (b) Quantification of MSOT signals showed statistically insignificant uptakes in both kidneys from healthy mice (*P*=0.956), demonstrating unimpaired renal functions.

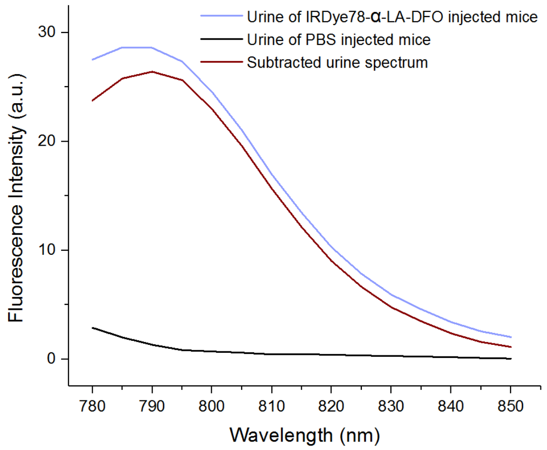


**Figure S22.** Raw fluorescence spectra for urine collected post *i.v.* injection of IRDye78-α-LA-DFO (blue) and PBS (black). IRDye78-α-LA-DFO spectrum is derived from subtraction (red).

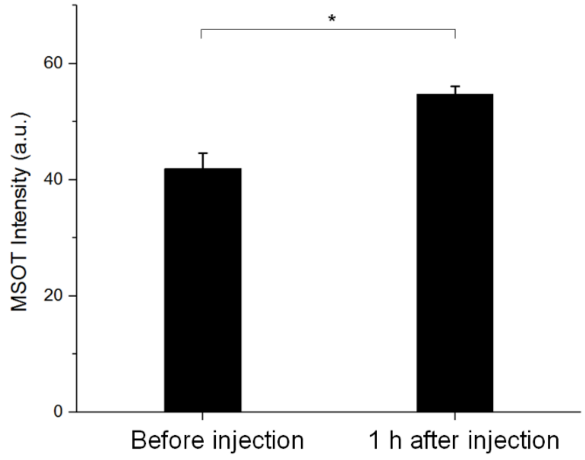


**Figure S23.** MSOT signal intensities in the tumorous area before and after injection (*P<0.05) in living mice bearing MDA-MB-231 tumors.

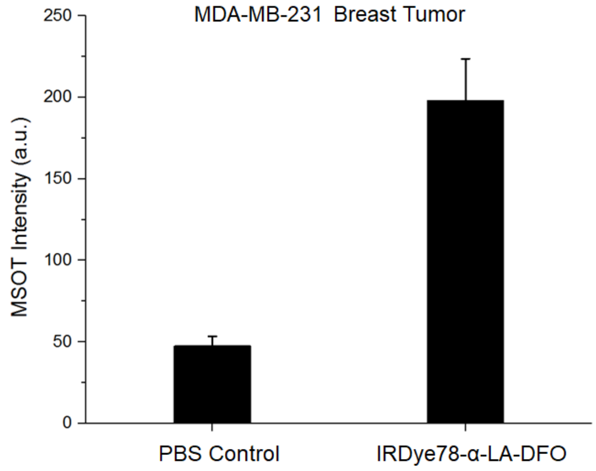


**Figure S24.** Multispectrally unmixed MSOT signals of breast tumors resected from xenograft mice 1 h following *i.v.* injection of IRDye78-α-LA-DFO in contrast to PBS.

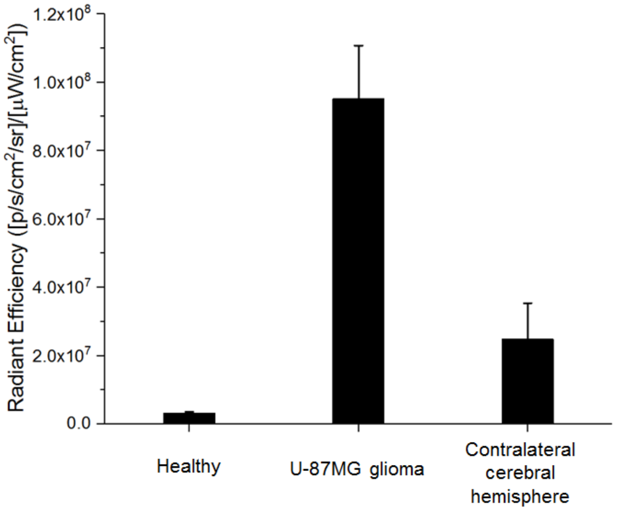


**Figure S25.** Quantification of ex vivo fluorescence signals in healthy brain, U-87MG glioma and the contralateral cerebral hemisphere.

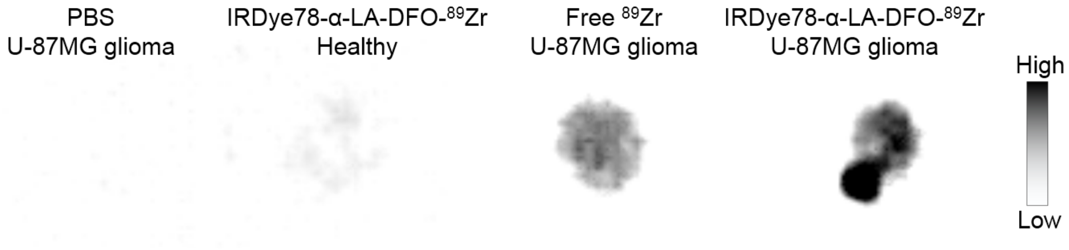


**Figure S26.** Ex vivo axial PET images of resected brains from mouse models of U-87MG glioma injected with PBS, free $Zr^{89}$ and IRDye78-α-LA-DFO-$Zr^{89}$. A healthy mouse with an intact blood-brain barrier was referenced to as a control.
